# Design and synthesis of natural antibacterial derivatives for ocular tuberculosis: a comprehensive review

**DOI:** 10.3389/fphar.2025.1697986

**Published:** 2025-11-20

**Authors:** Isaiah Osei Duah, Gabriel Amankwah, Josephine Ampong, Sherlene Brown, Bernice Lebene Tettey, Hubert Osei Acheampong, Ruth Boahemaa Awuku, Cynthia Amaning Danquah

**Affiliations:** 1 Department of Optometry and Visual Science, College of Science, Kwame Nkrumah University of Science and Technology, Kumasi, Ghana; 2 Department of Biological Sciences, College of Science, Purdue University, West Lafayette, IN, United States; 3 Department of Psychology, College of Health and Human Sciences, North Carolina Agricultural and Technical State University, Greensboro, NC, United States; 4 Department of Chemistry, College of Science, Kwame Nkrumah University of Science and Technology, Kumasi, Ghana; 5 Department of Chemistry, College of Science, Purdue University, West Lafayette, IN, United States; 6 Nesvard Institute of Molecular Sciences, Accra, Ghana; 7 Department of Internal Medicine, University of Texas Medical Branch, Galveston, TX, United States; 8 Graduate Program in Anatomy and Cell Biology, Wayne State University School of Medicine, Detroit, MI, United States; 9 Department of Family and Consumer Science, North Carolina Agricultural and Technical State University, Greensboro, NC, United States; 10 Department of Pharmacology, Faculty of Pharmacy and Pharmaceutical Sciences, College of Health Sciences, Kwame Nkrumah University of Science and Technology, Kumasi, Ghana

**Keywords:** ocular tuberculosis, natural antibacterial compounds, antibacterial drug design, drug delivery systems, medicinal chemistry, *Mycobacterium tuberculosis*

## Abstract

Ocular tuberculosis (TB) is an underrecognized extrapulmonary manifestation of *Mycobacterium tuberculosis (M. tuberculosis)* infection that can result in irreversible vision loss. Current systemic therapies, including isoniazid, rifampicin, pyrazinamide, and ethambutol, are often inadequate in achieving therapeutic intraocular concentrations and may pose ocular toxicity risks. The eye’s unique anatomical and physiological barriers, including the cornea, blood–aqueous, and blood–retinal barriers, limit drug penetration, particularly to the posterior segment. This paper explores the potential of natural antibacterial compounds as candidates for ocular TB therapy, emphasizing on rational drug design, chemical modification, and targeted drug delivery. Phytochemicals such as, plant-derived alkaloids, flavonoids, terpenoids, quinone, polyphenols, and saponins offer promising antibacterial scaffolds, which can be optimized for ocular bioavailability and safety through structural modification, prodrug strategies, and hybridization with other bioactive moieties. Advanced drug delivery systems, including nanoparticles, liposomes, nanogels, sustained-release implants, and *in situ* gelling systems, can overcome ocular barriers and maintain therapeutic drug concentrations. Preclinical evaluation using *in vitro*, *ex vivo*, and *in vivo* ocular models is critical to assess antimicrobial efficacy, pharmacokinetics, and toxicity. Clinical translation requires careful integration with systemic therapy, robust trial design, and navigation of regulatory frameworks, with particular attention to resource-limited settings. Future directions include computational modeling, personalized therapy, and global accessibility to ensure equitable implementation. By combining natural product chemistry, innovative drug delivery strategies, and translational research, next-generation ocular TB therapies have the potential to prevent vision loss and improve patient outcomes worldwide.

## Graphical Abstract

**Figure:**
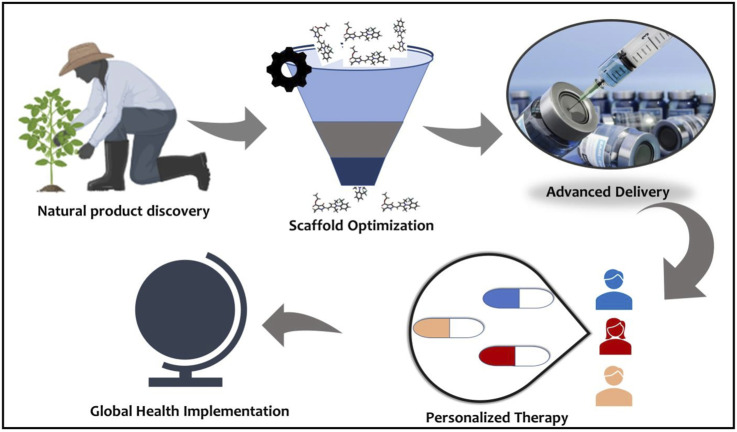

## 1 Introduction

Tuberculosis (TB) remains a major global health challenge despite decades of biomedical progress (Sizemore et al., 2012). In 2022, over 10 million new TB cases were reported worldwide, resulting in approximately 1.3 million deaths (Bagcchi, 2023). While pulmonary TB dominates the clinical landscape, extrapulmonary manifestations, including ocular TB are significant yet underrecognized (Akkerman et al., 2023; Gupta V. et al., 2015). It is particularly insidious, often progressing silently until irreversible visual impairment occurs (Albert and Raven, 2017), with the greatest burden in low- and middle-income countries where high TB prevalence coincides with limited diagnostic and therapeutic resources (Agrawal et al., 2018; Testi et al., 2020).

Ocular TB arises when *M. tuberculosis* infects ocular tissues, most commonly via hematogenous spread from a primary pulmonary or extrapulmonary focus, although rare cases involve direct extension from adjacent structures such as the sinuses or orbit (Basu, 2022; Basu et al., 2015; Madge et al., 2008). Disease progression is driven by both microbial activity and host immune responses (Sen, 2017; Sharma et al., 2011). Granulomatous inflammation, mediated by T-helper 1 lymphocytes and macrophages, forms the histopathological hallmark of ocular TB (Basu et al., 2015; Sharma et al., 2011). While granulomas may contain bacilli in a latent state, uncontrolled inflammation can provoke chronic ocular disease. Hypersensitivity reactions to mycobacterial antigens further exacerbate tissue damage, complicating diagnosis and treatment (Basu et al., 2020; Sen, 2017; Sharma et al., 2011).

**FIGURE 1:**
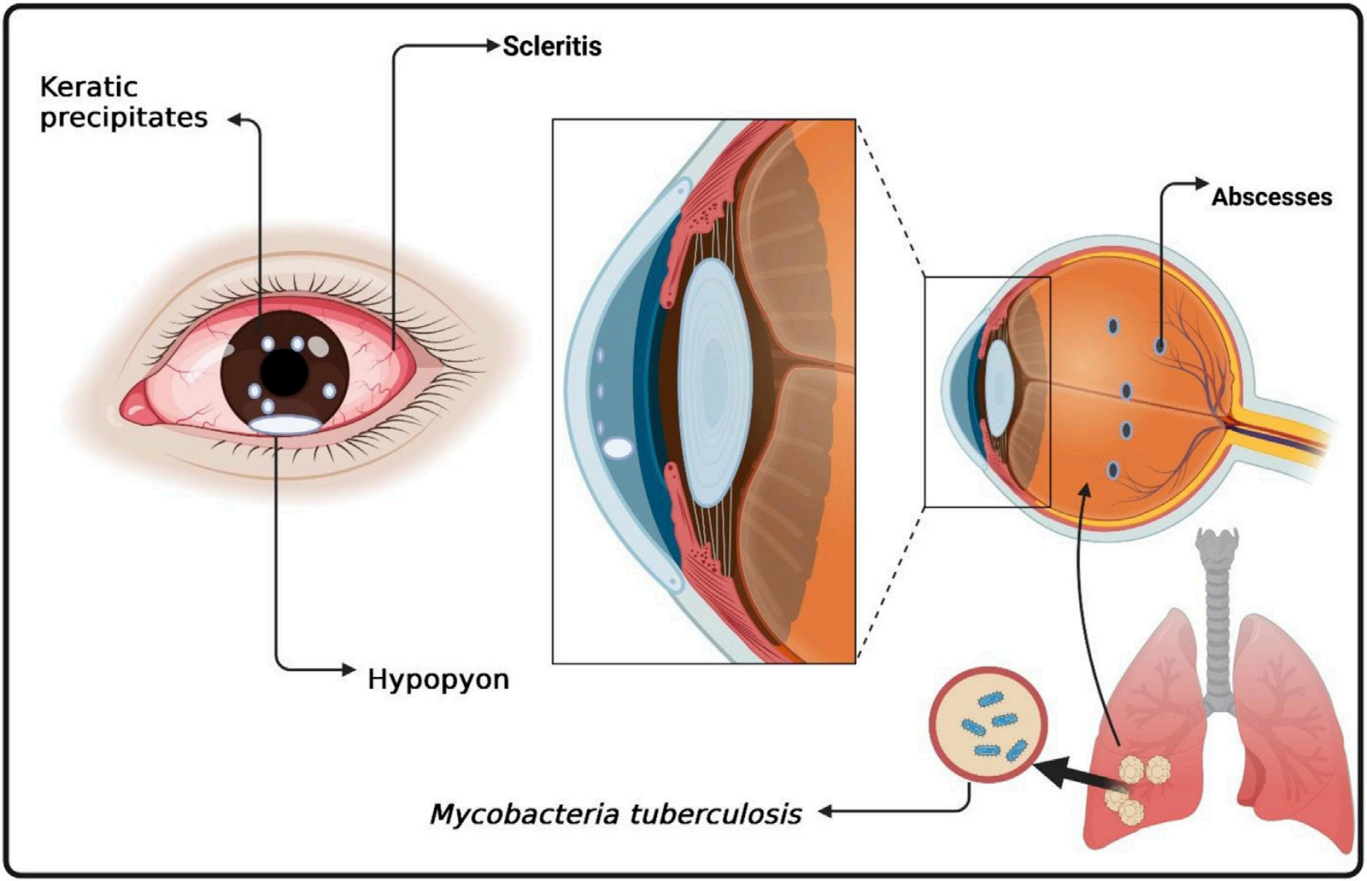
Anatomical and Pathophysiological Features of Ocular Tuberculosis. A cross-sectional schematic of the eye illustrates the cornea, anterior chamber, lens, vitreous, retina, and choroid. The uvea, choroid, and retina―sites commonly affected by ocular tuberculosis are highlighted. Arrows depict the hematogenous spread of Mycobacterium tuberculosis from the lungs to the eye, while icons represent granulomatous inflammation and immune cell infiltration.

Of note, ocular TB is defined primarily by clinical criteria, which include characteristic ocular signs, ancillary evidence of systemic TB infection (immunological or radiological tests), and the exclusion of non-TB etiologies rather than requiring histopathological or microbiological confirmation (Basu, 2022; Gupta A. et al., 2015). Clinically, ocular TB presents with a wide spectrum of manifestations affecting both the anterior and posterior segments of the eye. Anterior segment involvement commonly includes granulomatous uveitis, keratic precipitates, and posterior synechiae, whereas scleritis and interstitial keratitis occur less frequently (Sulaiman et al., 2024). Posterior segment disease may present as choroidal tubercles, choroiditis, retinal vasculitis, and neuroretinitis, with macular lesions threatening central vision (Sharma et al., 2011) see (Figure 1: Anatomical and Pathophysiological Features of Ocular TB). Rarely, severe presentations such as endophthalmitis and panophthalmitis occur. The variability and subtlety of early disease complicate timely diagnosis, which relies on ocular imaging, immunological assays, systemic TB evaluation, and therapeutic response (Sharma et al., 2011).

Current systemic TB therapies, including isoniazid, rifampicin, pyrazinamide, and ethambutol, are life-saving but often fail to achieve therapeutic concentrations in ocular tissues, particularly the posterior segment (Gupta et al., 2011). Certain agents, notably ethambutol and linezolid, carry ocular toxicity risks such as optic neuropathy, while multidrug-resistant TB further limits effective options (Aggarwal et al., 2021; Libershteyn, 2016). Anatomical barriers such as the cornea, blood–aqueous barrier, and blood–retinal barrier restrict drug penetration (Gunda et al., 2008), highlighting the urgent need for innovative ocular therapies.

Natural antibacterial compounds present a promising avenue for next-generation ocular TB treatment (Maiolini et al., 2020). Plant-derived molecules, including alkaloids, flavonoids, terpenoids, polyphenols, and saponins, exhibit diverse antimycobacterial activity, such as disruption of cell wall integrity and inhibition of key bacterial enzymes, and often demonstrate favorable biocompatibility, potentially reducing ocular toxicity (Bhat et al., 2018; Maiolini et al., 2020). Rational drug design enables the selection of bioactive molecules based on structure–activity relationships, while chemical modification can enhance solubility, stability, and ocular penetration through prodrug formation or functional group derivatization (Nunes et al., 2020). Targeted drug delivery strategies such as nanoparticles, liposomes, and hydrogels can overcome ocular barriers, achieve sustained intraocular concentrations, improve tissue retention, and minimize systemic exposure (Arabpour et al., 2024; Jacob et al., 2022; Nair et al., 2023).

By integrating rational drug design, medicinal chemistry, and advanced drug delivery, natural antibacterial compounds may be transformed into effective ocular TB therapeutics capable of reaching challenging intraocular sites, reducing toxicity, and ultimately preserving vision in affected patients (Pasqualoto and Ferreira, 2001). Specifically, natural compounds provide a unique starting point, offering structural diversity, multitarget activity, and inherent biocompatibility that are highly relevant for treating ocular TB (Maiolini et al., 2020). By leveraging rational drug design, medicinal chemistry, and scaffold optimization, these molecules can be transformed into more potent derivatives with improved ocular penetration, stability, and sustained activity against *M. tuberculosis* (Breijyeh and Karaman, 2023; Prajapati et al., 2024). Advanced delivery strategies—including nanoparticles, liposomes, cyclodextrins, and dendrimers—further expand the therapeutic window by overcoming ocular barriers and enabling targeted delivery to posterior segment tissues (Gabai et al., 2023; Wang et al., 2021). Future integration of computational modeling, structure–activity relationship studies, and green synthetic approaches will accelerate the translation of natural product scaffolds into next-generation ocular therapeutics (Gangwal and Lavecchia, 2025). Ultimately, the strategic development of natural antibacterial compounds tailored for intraocular use represents a promising frontier in combating extrapulmonary TB, preserving vision, and enhancing patient outcomes. To this end, this review aims to explore the potential of natural antibacterial compounds as candidates for ocular TB therapy, emphasizing rational drug design, chemical modification, and targeted drug delivery to overcome current limitations in systemic treatment. Taken together, augmenting the pipeline of anti-tubercular ocular drugs holds promise not only for preventing vision loss but also for improving overall quality of life in affected patients.

## 2 Limitations of current therapy and the potential of natural antibacterial compounds

Systemic anti-tubercular therapy remains the standard of care for ocular TB, typically following the four-drug regimen of isoniazid, rifampicin, pyrazinamide, and ethambutol (Betzler et al., 2023; Kee et al., 2016). While these agents are highly effective against pulmonary and systemic TB, their efficacy in ocular TB is often suboptimal (Sabhapandit et al., 2023). The unique anatomy and physiology of the eye create formidable barriers to drug penetration. The corneal epithelium, conjunctiva, and tight junctions of the blood–aqueous and blood–retinal barriers limit the diffusion of hydrophilic and large molecular weight compounds, resulting in sub-therapeutic concentrations in intraocular tissues, particularly in the posterior segment (Ramsay et al., 2018; Ramsay et al., 2019; Toda et al., 2011). Consequently, even prolonged systemic therapy frequently fails to achieve therapeutic intraocular levels, leading to persistent or recurrent ocular inflammation (Multani et al., 2022).

In addition to inadequate ocular penetration, several standard TB drugs carry intrinsic risks of ocular toxicity (Aggarwal et al., 2021). Ethambutol is well-known for dose-related optic neuropathy, manifesting as central visual loss, color vision deficits, and optic disc pallor (Sabhapandit et al., 2023). Linezolid, increasingly employed for multidrug-resistant TB, similarly poses optic nerve toxicity with prolonged use (Aljebreen et al., 2020). Rifabutin may provoke uveitis in susceptible individuals (Saux et al., 1997). These adverse effects complicate clinical management, as distinguishing drug-induced ocular injury from disease progression is challenging.

Treatment duration and multidrug resistance further exacerbate these limitations. Drug-sensitive TB requires at least 6 months of therapy, whereas multidrug-resistant TB regimens may extend beyond 18 months (Karnan et al., 2024). Long-term systemic treatment increases cumulative toxicity and challenges adherence, particularly in resource-limited settings. Resistance to cornerstone drugs like isoniazid and rifampicin reduces the efficacy of standard regimens and necessitates second-line agents that often fail to achieve adequate ocular levels and carry systemic toxicities, including nephrotoxicity, hepatotoxicity, and hematologic complications (Gegia et al., 2017; Prasad et al., 2021). Adding to these challenges, rifampicin has been shown to upregulate MDR1/P-glycoprotein (P-gp) expression and activity in human THP-1–derived macrophages, leading to enhanced drug efflux and reduced intramacrophage accumulation of prothionamide, a P-gp substrate (Hasanuzzaman et al., 2019). This mechanism may further compromise the intracellular efficacy of several antitubercular agents. Together, these challenges underscore the urgent need for innovative approaches to ocular TB therapy, and natural antibacterial compounds present a promising avenue. Plant-derived molecules, including alkaloids, flavonoids, terpenoids, polyphenols and saponins, offer unique advantages for ocular application (Koirala et al., 2021; Kumar et al., 2021). Their chemical diversity allows them to target multiple bacterial pathways, such as cell wall synthesis, efflux pump function, nucleic acid metabolism, and key enzymatic processes, often with a lower risk of resistance development (Koirala et al., 2021; Mallik et al., 2025). Many also demonstrate inherent biocompatibility, reducing the potential for ocular toxicity compared to conventional TB drugs (Anand et al., 2019).

The structural versatility of natural compounds enables medicinal chemistry optimization to enhance solubility, tissue penetration, and antimicrobial potency (Mazlun et al., 2019). Alkaloids like berberine and piperine exhibit antimycobacterial activity and can be modified for improved ocular delivery (Ozturk et al., 2021; Sarangi et al., 2021). Flavonoids such as quercetin and luteolin not only inhibit mycobacterial enzymes but also possess anti-inflammatory properties, potentially mitigating immune-mediated tissue damage (Cetin, 2021). Terpenoids, including ursolic acid and betulinic acid, disrupt bacterial membranes and may act synergistically with other agents (Jagatap et al., 2022; Jiménez-Arellanes et al., 2013), while polyphenols and tannins provide bacteriostatic effects suitable for combination therapy (Prusty et al., 2024).

Crucially, natural compounds can be integrated into advanced ocular drug delivery systems. Encapsulation in nanoparticles, liposomes, or hydrogels facilitates controlled release and targeted deposition in both anterior and posterior ocular compartments (Arabpour et al., 2024; Nair et al., 2023). Prodrug strategies can further enhance corneal permeability and tissue retention while minimizing systemic exposure (Taskar et al., 2017). The combination of antimicrobial potency, structural adaptability, and favorable safety profiles positions natural products as ideal candidates for next-generation ocular TB therapy, capable of overcoming the limitations of conventional systemic regimens and preventing vision loss.

## 3 Natural compound selection, mechanisms, and scaffold design

**FIGURE 2:**
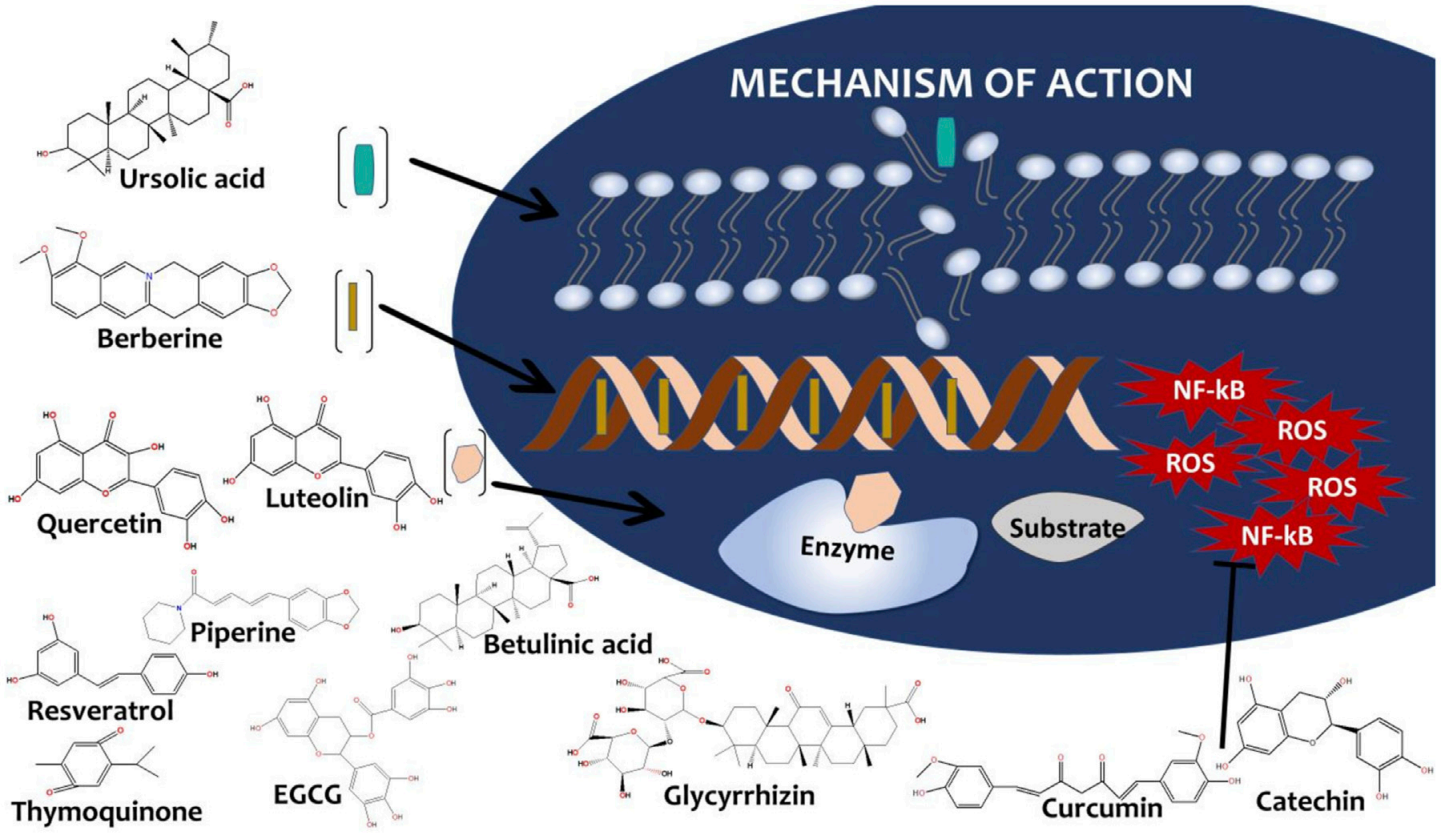
Natural Compounds and Mechanisms of Action. Molecular schematic and mechanistic illustration prepared with ChemDraw, showing representative natural compounds with antimycobacterial potential. Key structures include alkaloids (berberine, piperine), flavonoids (quercetin, luteolin, EGCG), terpenoids (ursolic acid, betulinic acid), polyphenols (curcumin, catechin, resveratrol), quinones (thymoquinone), and saponins (glycyrrhizin). Mechanistic icons highlight their primary modes of action, including DNA intercalation (berberine), enzyme inhibition (quercetin, luteolin), membrane disruption (ursolic acid), and anti-inflammatory/antioxidant effects (curcumin, catechin).

Natural products have long served as a rich source of antimicrobial agents. Their structural diversity, evolved biological activity, and favorable biocompatibility make them particularly attractive for Ocular TB therapy (Nguta et al., 2015; Quan et al., 2017). Rational selection of natural compounds begins with identifying molecules with proven or potential antimycobacterial activity and suitable physicochemical properties for ocular penetration (Nunes et al., 2020). The key classes of natural antibacterial derivatives include but not limited to alkaloids, flavonoids, terpenoids, quinone, polyphenols, and saponins (Patra, 2012) that elicits anti-tubercular effect through DNA intercalation, enzyme inhibition, membrane disruption, anti-inflammatory and anti-oxidant activity as seen in Figure 2: Natural compounds and mechanism of action.

### 3.1 Alkaloid derivatives

Alkaloids are nitrogen-containing compounds widely distributed in plants, exhibiting potent antimicrobial and immunomodulatory activity (Semmar, 2024). Berberine, extracted from *Berberis* species, exerts broad-spectrum antibacterial effects, including inhibition of *M. tuberculosis*, by intercalating into bacterial DNA and disrupting replication and transcription (Ozturk et al., 2021). Its cationic nature enhances interaction with negatively charged mycobacterial cell walls, promoting bactericidal activity (Zhou et al., 2023). In Ocular TB, berberine may mitigate retinal and choroidal oxidative damage, suppresses excessive inflammatory responses, and supports local immune control of intracellular bacilli (Ozturk et al., 2021). Liposomal and dendrimer-based formulations may enhance its intraocular bioavailability and retention, ensuring effective concentrations in anterior and posterior segments while minimizing systemic exposure (Akhter et al., 2022). However, berberine has been associated with gastrointestinal discomfort, nausea, and constipation, and at higher systemic doses, with potential hepatotoxicity and inhibition of cytochrome P450 enzymes (CYP2D6, CYP2C9, and CYP3A4), and multi-drug resistance protein 1 which may alter drug metabolism (Guo et al., 2012; Imenshahidi and Hosseinzadeh, 2019). Additional toxic effects include impaired hepatic ammonia detoxification, reduced liver ATP levels, an increased NADH/NAD^+^ ratio, and mitochondrial dysfunction with impaired pyruvate carboxylation (Moreira et al., 2022). Piperine, derived from *Piper nigrum*, inhibits efflux pumps in mycobacteria, increasing intracellular antimicrobial retention and potentiating synergy with other compounds (Sharma et al., 2010). Structural modifications, including methylation or esterification, improve lipophilicity and facilitate corneal and posterior segment penetration, enhancing therapeutic efficacy (Bhambhani et al., 2021). Nonetheless, piperine may cause gastrointestinal irritation, hepatotoxicity at higher doses, and alter pharmacokinetics of co-administered drugs through inhibition of cytochrome P450 enzymes and P-glycoprotein, raising concerns about potential drug–drug interactions (Lee et al., 2018; Shrestha et al., 2025).

### 3.2 Flavonoid derivatives

Flavonoids are polyphenolic compounds abundant in fruits and vegetables, with multiple mechanisms against *M. tuberculosis* and significant ocular protective effects (Mickymaray et al., 2020; Rabaan et al., 2022). Quercetin and luteolin inhibit bacterial enzymes such as DNA gyrase and enoyl-ACP reductase, while their anti-inflammatory activity reduces oxidative tissue damage (Aydin et al., 2025; Sasikumar et al., 2018). However, high doses of quercetin causes nausea, vomiting, sweating, flushing, dyspnea, and kidney toxicity (Lakhanpal and Rai, 2007; Mehjabin et al., 2024) whereas luteolin temporarily increase irritability in children with autism spectrum disorders (Taliou et al., 2013). Catechin and epigallocatechin gallate (EGCG), flavan-3-ols found in green tea, protect retinal pigment epithelium (RPE) and corneal cells from oxidative stress, modulate immune responses by suppressing cytokine overproduction, and mitigate tissue injury (Ng et al., 2023). Nanocarrier systems improve solubility, ocular bioavailability, and targeted delivery to posterior ocular segments, enabling sustained therapeutic effects. Catechin induce gastrointestinal symptoms such as nausea, vomiting, diarrhea, abdominal pain, bloating and potential liver toxicity (Navarro et al., 2013; Nawab and Farooq, 2015) whereas high doses of EGCG potentiate chemically-induced colitis (Martin and Bolling, 2015). Genistein and resveratrol provide antioxidant, anti-inflammatory, and immunomodulatory protection, reducing retinal and choroidal injury and enhancing ocular microvascular health (Delmas et al., 2021; Lançon et al., 2016); nanoparticle formulations further improve tissue penetration and retention (Krstić et al., 2021). Naringenin exerts similar protective effects, including inhibition of angiogenesis and prevention of retinal degeneration, with cyclodextrin or chitosan-based nanoparticles optimizing delivery (Bhia et al., 2021; Zucca et al., 2025). Despite these benefits, genistin has the potential to slow caffeine metabolism through decreases in CYP1A2 and increase in CYP2A6 activity (Chen et al., 2011), and impair ovarian differentiation and estrous cyclicity at therapeutic doses (Guelfi et al., 2023). Kaempferol and baicalin offer antioxidant, anti-inflammatory, and anti-fibrotic effects, protecting the macula, retina, and cornea from immune-mediated injury, while nanoparticle carriers enhance bioavailability, stability, and sustained release (Kaabi, 2022). Of note, kaempferol interaction with iron, reduces its absorption and potentially causes detrimental effects in individuals with iron deficiency (Hu et al., 2006; Milman, 2020) whereas high doses of baicalin can induce kidney injury and fibrosis (Cai et al., 2017).

### 3.3 Terpenoid derivatives

Terpenoids, composed of isoprene units, exhibit potent antimycobacterial and tissue-protective effects (Jagatap et al., 2022). Ursolic acid and betulinic acid disrupt bacterial membranes, increasing permeability and inducing cell death (Farzan et al., 2024; Oloyede et al., 2017). Their lipophilic nature favors corneal absorption, but nanoencapsulation ensures sustained release, reduces local irritation, and improves ocular pharmacokinetics (Ahmed et al., 2023; Bhandari et al., 2022). While generally considered safe, long-term or sub-acute oral administration of betulinic acid and ursolic acid causes mild toxicity, including liver and kidney stress (elevated serum glutamic-oxaloacetic transaminase [SGOT], alkaline phosphatase [ALP], and urea), hematological changes, and histopathological alterations in the liver, kidney, and spleen (Mishra et al., 2021). Tetrandrine, an alkaloid-terpenoid hybrid, demonstrates anti-inflammatory, immunomodulatory, and anti-fibrotic activity (Li et al., 2024). In ocular TB, it reduces neovascularization, prevents posterior capsule fibrosis, and limits inflammatory tissue remodeling. As a calcium channel blocker, tetrandrine lowers blood pressure, which may result in hypotension accompanied by dizziness, fainting, blurred vision, or even shock; therefore, caution is advised in patients with cardiovascular conditions (Man-Ren, 2002). Delivery of terpenoids via solid lipid nanoparticles enhances tissue accumulation, stability, and intraocular retention, maximizing therapeutic outcomes (Jacob et al., 2022; Lasoń, 2020). Topical use of terpenoids induce ocular irritation and allergic reactions in sensitive individuals, and their poor water solubility and stability can further limit clinical application (Baptista-Silva et al., 2020; Gajbhiye and Pal, 2024).

### 3.4 Quinone derivatives

Thymoquinone (TQ), a quinone derived from *Nigella sativa*, combines neuroprotective, antioxidant, anti-inflammatory, and anti-fibrotic activities (Sadeghi et al., 2023). In ocular TB, TQ protects retinal and corneal tissues from oxidative and inflammatory damage, modulates pro-inflammatory cytokine production, and inhibits fibrotic pathways, reducing scarring and tissue remodeling (Hannan et al., 2021; Hu et al., 2019; Mahmud et al., 2022). Its multi-target activity suggests potential as an adjunct therapy in TB-related ocular complications (Hu et al., 2019). Further, members of the vitamin K group, structurally characterized as 2-methyl-1,4-naphthoquinone derivatives, exhibit notable activity against mycobacteria (Bashiri et al., 2020; Chadar et al., 2015). This is not unexpected, as these compounds share a common naphthoquinone core with rifampicin, a cornerstone antitubercular agent (Ganapathy et al., 2021; Halicki et al., 2018). The chemical similarity between vitamin K derivatives and rifampicin may underlie their antimycobacterial properties, suggesting that naphthoquinone scaffolds can serve as a promising basis for designing new antitubercular compounds (Ganapathy et al., 2021; Halicki et al., 2018). While generally considered safe, acute high doses can cause hypoactivity, respiratory difficulty, oxidative stress, and biochemical signs of liver, kidney, and heart stress (Badary et al., 1998).

### 3.5 Polyphenol derivatives

Curcumin, a polyphenol from *Curcuma longa*, shows strong anti-inflammatory, antioxidant, and immunomodulatory properties that make it highly relevant for ocular TB. In TB-associated uveitis and choroiditis, excessive granulomatous inflammation drives retinal and choroidal injury. Curcumin attenuates this response by suppressing pro-inflammatory cytokines tumor necrosis factor-alpha [(TNF-α) interluekin (IL)-1β, IL-6] and enhancing anti-inflammatory cytokine IL-10 (Kumar et al., 2017; Mollazadeh et al., 2019). Central to this effect is inhibition of nuclear factor kappa-light-chain-enhancer of activated B cells (NF-κB) signaling, a key pathway in immune activation and fibrosis (Leclercq et al., 2004). By limiting nuclear factor kappa-light-chain-enhancer of activated B cells (NF-κB) activity, curcumin reduces macrophage and T-cell recruitment to granulomas, thereby minimizing collateral tissue damage (Memarzia et al., 2021). In addition to immune regulation, curcumin protects RPE and lens epithelial cells from oxidative stress, preserving retinal integrity and preventing cataract formation (Mandal et al., 2009). Its antioxidant activity,via scavenging reactive oxygen species and activating Nuclear factor erythroid 2–related factor 2 (Nrf2) pathways, further reduces tissue injury during chronic inflammation (Ashrafizadeh et al., 2020). Despite these benefits, curcumin’s poor aqueous solubility and low ocular bioavailability restrict therapeutic use (Ribeiro et al., 2025). Advances in drug delivery systems such as cyclodextrin complexes, liposomes, and nanoparticles improve its solubility, stability, and intraocular penetration, enabling sustained release and enhanced efficacy (Li et al., 2017).

Although curcumin is generally considered safe at dietary doses, but high systemic or long-term intake may cause gastrointestinal symptoms such as nausea, diarrhea, and abdominal discomfort (Akyakar et al., 2025; Hewlings and Kalman, 2017). Rarely, it can induce hepatotoxicity or interact with anticoagulants and antiplatelet agents, potentially increasing bleeding risk. Curcumin may also affect drug metabolism by inhibiting certain cytochrome P450 enzymes, altering the pharmacokinetics of co-administered medications (Appiah-Opong et al., 2007; Keihanian et al., 2018; Parvez and Anjum, 2022; Thapliyal and Maru, 2001). Overall, curcumin represents a promising adjunct therapy in ocular TB. By reducing granulomatous inflammation, protecting ocular cells, and improving vision preservation, it complements conventional anti-TB drugs while minimizing long-term ocular morbidity (Franzone et al., 2021; Ribeiro et al., 2025). Other polyphenols, including catechins, ellagic acid, and gallic acid, inhibit mycobacterial cell wall synthesis and protein function while providing antioxidant protection to inflamed ocular tissues (Caban et al., 2022; Krstić et al., 2021). Scaffold modifications, such as esterification or conjugation to hydrophilic carriers, enhance solubility and ocular tissue penetration (Biswas et al., 2024; Shastri et al., 2023).

### 3.6 Saponin derivatives

Saponins, glycosidic compounds found in numerous plants, offer both direct antimicrobial activity and immune modulation (Brindha, 2016; Pikhtirova et al., 2023). Certain saponins can disrupt bacterial membranes while stimulating host immune responses, potentially enhancing clearance of intracellular bacilli within ocular macrophages (Pikhtirova et al., 2023). Chemical optimization can improve ocular stability and reduce hemolytic activity, facilitating safe ocular administration. However, saponins can still induce local irritation, transient inflammation, or cytotoxicity at high concentrations (Tizard, 2020), and systemic absorption may lead to hemolysis or gastrointestinal disturbances (Oakenfull and Sidhu, 2023).

Synthetic and semi-synthetic strategies enable optimization of these natural scaffolds (Moses et al., 2014). Prodrug approaches, in which active compounds are masked by ester or amide groups and activated by ocular enzymes, can enhance corneal permeability and intraocular retention (Kumar et al., 2014). Hybrid molecules, combining complementary scaffolds such as flavonoid-alkaloid conjugates may target multiple bacterial pathways simultaneously, reducing the likelihood of resistance (Jayaraman et al., 2013; Si et al., 2023). Nonetheless, chemical modifications can introduce off-target toxicity, hypersensitivity reactions, or metabolic instability, necessitating careful preclinical evaluation(Marrelli et al., 2016; Thakur et al., 2013).

Green chemistry techniques and click chemistry approaches allow modular synthesis of derivatives with tunable lipophilicity, polarity, and molecular weight, optimizing both antimicrobial potency and ocular pharmacokinetics (Bishnoi et al., 2025; Shirame and Bhosale, 2018).

Computational modeling can further guide scaffold optimization (Taldaev et al., 2022). Molecular docking predicts interactions with bacterial enzymes, while physicochemical modeling estimates permeability across ocular barriers (de Oliveira et al., 2021; Singh and Shukla, 2012). Structure-activity relationship (SAR) studies inform rational modifications that maximize potency while minimizing toxicity. For example, modification of hydroxyl or methoxy groups on flavonoid cores can enhance membrane penetration without compromising antibacterial activity (Shamsudin et al., 2022; Xie et al., 2015).

Ultimately, scaffold design integrates multiple considerations: antimicrobial efficacy, ocular tissue penetration, biocompatibility, stability, and compatibility with advanced delivery systems. By strategically selecting and modifying natural compounds, it is possible to create a new generation of ocular TB drugs capable of achieving therapeutic concentrations in challenging ocular compartments while maintaining safety. Summary of natural compounds their mechanism of action and potential toxicity are highlighted in Table 1.

**TABLE 1:** Natural compounds for ocular TB.

Compound class	Compound	Mechanism of action	Study type	Key findings	Toxicity/Limitations
Alkaloids	Berberine	DNA intercalation, inhibition of replication/transcription; interacts with mycobacterial cell wall	*In vitro,* *in vivo*	Broad-spectrum antimycobacterial activity; reduces oxidative stress and inflammation in retinal/choroidal tissue	GI discomfort, constipation; hepatotoxicity at high doses; CYP450 inhibition; mitochondrial dysfunction
	Piperine	Efflux pump inhibition; enhances intracellular antimicrobial retention	*In vitro*	Potentiates synergy with other antimicrobials; improved ocular penetration with structural modifications	GI irritation, hepatotoxicity at high doses; drug–drug interactions via CYP450 and P-gp
Flavonoids	Quercetin	DNA gyrase and enoyl-ACP reductase inhibition; anti-inflammatory	*In vitro,* *in vivo*	Reduces oxidative tissue damage; supports immune control of *M. tuberculosis*	High doses: nausea, vomiting, kidney toxicity
	Luteolin	Enzyme inhibition, anti-inflammatory	*In vitro*	Protects retinal and corneal tissue	May increase irritability in children with ASD
	Catechin/EGCG	Antioxidant, immune modulation; protects RPE and cornea	*In vitro,* *in vivo*	Reduces oxidative stress; improves intraocular bioavailability with nanocarriers	GI disturbances; EGCG may worsen chemically induced colitis
	Genistein/Resveratrol	Antioxidant, anti-inflammatory, immunomodulatory	*In vitro*	Reduces retinal/choroidal injury; supports ocular microvasculature	May affect CYP enzymes; impact on ovarian function at high doses
	Kaempferol/Baicalin	Antioxidant, anti-fibrotic	*In vitro*	Protects macula, retina, cornea; enhanced delivery with nanoparticles	Kaempferol: iron absorption interference; Baicalin: kidney injury at high doses
	Naringenin	Anti-angiogenic, retinal protection	*In vivo*	Prevents retinal degeneration; optimized with nanoparticles	Limited systemic toxicity data
Terpenoids	Ursolic acid/Betulinic acid	Disrupts bacterial membranes; lipophilic enhances corneal absorption	*In vitro,* *in vivo*	Antimycobacterial; tissue-protective; nanoencapsulation improves ocular retention	Mild hepatotoxicity, kidney stress, hematologic changes with long-term use
	Tetrandrine	Anti-inflammatory, immunomodulatory, anti-fibrotic; calcium channel blocker	*In vivo*	Reduces neovascularization, posterior capsule fibrosis, tissue remodeling	Hypotension, dizziness, blurred vision; caution in cardiovascular disease
Quinones/Polyphenols	Thymoquinone (TQ)	Neuroprotective, antioxidant, anti-inflammatory, anti-fibrotic	*In vitro,* *in vivo*	Protects retinal/corneal tissue; reduces inflammation and fibrosis	High doses: hypoactivity, respiratory difficulty, liver/kidney/heart stress
	Curcumin	Anti-inflammatory, antioxidant, NF-κB inhibition	*In vitro,* *in vivo*, clinical	Reduces granulomatous inflammation; protects RPE and lens; supports vision preservation	Poor aqueous solubility; high doses: GI symptoms, rare hepatotoxicity, anticoagulant interactions
	Catechins, Ellagic acid, Gallic acid	Inhibit mycobacterial cell wall/protein synthesis; antioxidant	*In vitro*	Protects ocular tissue; supports bacterial clearance	Limited ocular toxicity data; solubility issues
Saponins	Saponins (general)	Membrane disruption; immune stimulation	*In vitro,* *in vivo*	Enhance clearance of intracellular bacilli; stimulate host immunity	Local irritation, transient inflammation, cytotoxicity at high concentrations; systemic hemolysis/GI disturbances
	Hybrid/Prodrug Saponins	Multi-target bacterial pathway inhibition; improve ocular penetration	*In vitro*	Strong multi-target action; better intraocular retention	Potential off-target toxicity; metabolic instability

Of note, combining natural compounds with complementary mechanisms can produce synergistic effects, enhancing antimicrobial efficacy and improving ocular TB therapy. Flavonoids such as quercetin and luteolin inhibit bacterial DNA gyrase and enoyl-ACP reductase (Gallegos et al., 2016) while simultaneously reducing oxidative stress in ocular tissues (Hytti et al., 2015; Li et al., 2021; Lu et al., 2015). Terpenoids like ursolic acid and betulinic acid disrupt mycobacterial membranes, facilitating enhanced intracellular delivery of other compounds (Ahmed et al., 2023; Bhandari et al., 2022), and saponins further increase intracellular bioavailability while stimulating host immune defenses, collectively targeting multiple bacterial pathways (Farzan et al., 2024; Francis et al., 2002; Oloyede et al., 2017). This multi-target approach reduces the likelihood of resistance, lowers the required doses of individual compounds, and minimizes potential toxicity. Natural compounds can also be co-administered with conventional antitubercular drugs, including isoniazid, rifampicin, and ethambutol, to improve therapeutic outcomes. Certain flavonoids and terpenoids enhance drug penetration across ocular barriers, while others inhibit bacterial efflux pumps or protective enzymes, sensitizing *Mycobacterium tuberculosis* to standard therapy (Chen et al., 2016; Rabaan et al., 2022; Sharma et al., 2020). Additionally, compounds with anti-inflammatory and antioxidant properties, such as curcumin and thymoquinone, mitigate drug-induced ocular toxicity and limit tissue damage caused by granulomatous inflammation (Ahmad et al., 2024; Liu et al., 2025). By suppressing pro-inflammatory cytokines, including TNF-α, IL‐1β, and IL-6, and promoting regulatory cytokines such as IL-10 (Memarzia et al., 2021; Mollazadeh et al., 2019), these agents protect the retina, choroid, and cornea while supporting bacterial clearance. Taken together, rationally designed synergistic strategies, whether combining multiple natural compounds or pairing them with conventional antitubercular agents, represent a promising therapeutic approach for ocular TB, offering the potential to optimize microbial eradication, minimize tissue injury, and improve overall treatment outcomes, highlighting an exciting area for future research and clinical translation. The synergistic effects of natural compounds, alone or in combination with standard TB drugs, are summarized in Table 2.

**TABLE 2:** Synergistic natural compound and Anti-TB formulations in ocular TB.

Combination	Mechanism of synergy	Study type	Key findings	Benefits/Notes
Quercetin + luteolin	Dual inhibition of bacterial DNA gyrase and enoyl-ACP reductase; antioxidant, anti-inflammatory	*In vitro,* *in vivo*	Enhanced bacterial killing; reduced oxidative damage in retina/choroid	Multi-target approach reduces resistance; lowers individual compound doses
Ursolic acid + betulinic acid	Membrane disruption + increased intracellular delivery of co-administered drugs	*In vitro,* *in* *vivo*	Improved mycobacterial clearance; enhanced ocular penetration	Supports nanoformulation strategies for sustained intraocular release
Saponins + flavonoids/terpenoids	Membrane permeabilization + immune stimulation	*In vitro*	Increased intracellular bioavailability; enhanced macrophage-mediated clearance	Multi-pathway targeting; reduced potential toxicity via lower doses
Curcumin + thymoquinone	Anti-inflammatory, antioxidant, immunomodulatory	*In vivo*	Reduced granulomatous inflammation; protection of RPE, retina, and cornea	Mitigates drug-induced ocular toxicity; improves tissue preservation
Natural compounds + isoniazid/rifampicin/ethambutol	Flavonoids/terpenoids enhance drug penetration; inhibit efflux pumps; suppress protective enzymes in *M. tuberculosis*	*In vitro,* *in vivo*, clinical	Increased antimicrobial efficacy; shorter time to bacterial clearance	Potential to reduce doses of conventional drugs; limits ocular tissue damage
Piperine + anti-TB drugs	Efflux pump inhibition + enhanced intracellular retention of drugs	*In vitro*	Increased intracellular drug levels and bactericidal activity	Potentiates synergy with standard therapy; may improve ocular drug bioavailability
Hybrid molecules (flavonoid-alkaloid conjugates)	Multi-target bacterial pathway inhibition	*In vitro*	Stronger antimicrobial activity than individual scaffolds	Reduced risk of resistance; optimized ocular tissue penetration

Anti-TB, drugs: Isoniazid/Rifampicin/Ethambutol.

## 4 Advanced ocular drug delivery systems for natural compounds

The eye presents unique challenges for drug delivery due to its complex anatomy, protective barriers, and dynamic fluid compartments (Cabrera et al., 2019; Cholkar et al., 2013; Patel et al., 2010). Topical administration, systemic therapy, and local injections must navigate the cornea, conjunctiva, sclera, vitreous, and retinal barriers to achieve therapeutic concentrations without inducing toxicity (Cholkar et al., 2013). For natural antibacterial compounds, which often exhibit moderate solubility, variable stability, and susceptibility to enzymatic degradation, advanced drug delivery strategies are essential to optimize ocular bioavailability (Ghosh et al., 2024). Topical administration remains the most convenient and patient-compliant route, particularly for anterior segment disease, yet conventional eye drops suffer from rapid tear turnover, limited corneal permeability, and poor retention (Gupta et al., 2021). *In situ* gelling systems can address these limitations by transitioning from liquid to gel upon exposure to temperature, pH, or ionic conditions in the ocular environment, allowing natural compounds such as flavonoids and terpenoids to remain longer on the ocular surface and release drug gradually (Almeida et al., 2014; Gilani et al., 2022). Nanocarriers provide additional solutions (Gilani et al., 2022). Liposomes composed of phospholipid bilayers encapsulate hydrophobic compounds like ursolic acid, improving solubility, stability, and cellular uptake (Agarwal et al., 2016; López-Cano et al., 2021; Mishra et al., 2011). Polymeric nanoparticles fabricated from biodegradable materials such as poly (lactic-co-glycolic acid) (PLGA) enable controlled release over days to weeks, sustaining drug levels in anterior and posterior ocular compartments (Nagarwal et al., 2009). Nanogels with high water content and tunable mesh size can encapsulate hydrophilic polyphenols such as quercetin, enhancing corneal penetration while minimizing irritation (Liu et al., 2016; Wu et al., 2023).

Posterior segment disease, including choroiditis, retinal vasculitis, and neuroretinitis, is particularly challenging because systemic therapy rarely achieves sufficient intraocular concentrations (Gupta et al., 2007). Localized delivery, such as intravitreal and periocular injections, allows direct deposition of natural compounds at the site of infection (Albert and Raven, 2017). Biodegradable implants composed of PLGA or natural polymers such as chitosan can provide sustained release of compounds such as berberine or luteolin over several weeks, reducing injection frequency and systemic exposure (Wang et al., 2024). Non-invasive alternatives for anterior and intermediate segment delivery include contact lens-based systems, in which hydrogel lenses gradually release natural compounds onto the ocular surface to enhance corneal absorption (Zhao et al., 2023). Electrospun nanofibers and mucoadhesive films offer similar sustained-release platforms with adjustable dosing and minimal patient discomfort (Desai et al., 2025; Pérez-González et al., 2019).

**FIGURE 3:**
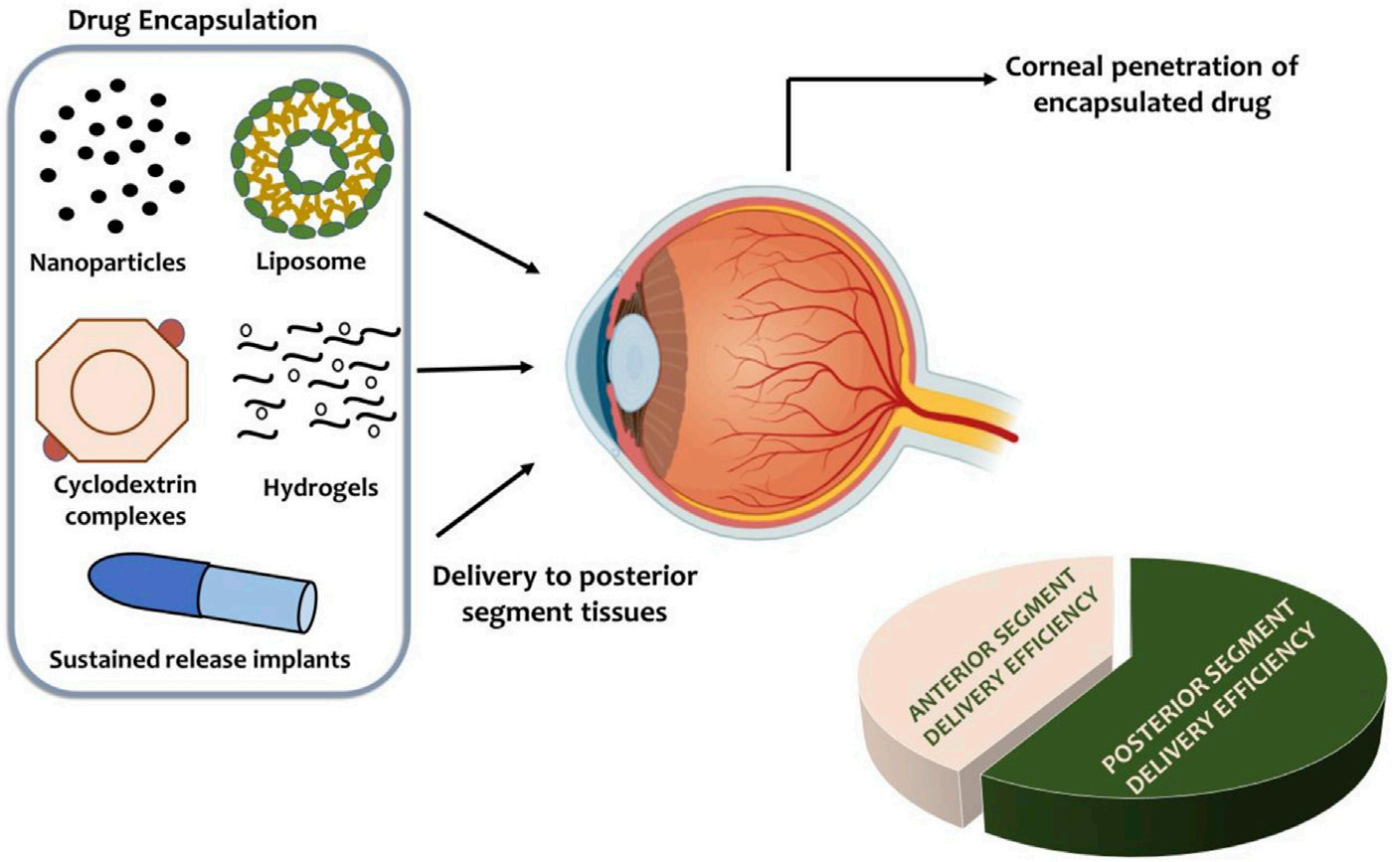
Advanced Ocular Drug Delivery Systems. Infographic schematic illustrating innovative ocular delivery platforms, including nanoparticles, liposomes, hydrogels, cyclodextrin complexes, and sustained- release implants. The diagram depicts drug encapsulation, corneal penetration, and transport to posterior segment tissues, with a comparative overlay highlighting differences in delivery efficiency to the anterior versus posterior segments.

Formulation strategies must consider the physicochemical properties of natural compounds. Hydrophobic compounds may require solubilization via cyclodextrins or lipid-based carriers (Farid et al., 2017), while compounds prone to enzymatic degradation can benefit from prodrug approaches in which esterified derivatives are hydrolyzed in ocular tissues to release the active molecule (Taskar et al., 2017). Surface modification of nanoparticles with targeting ligands such as mannose or transferrin can enhance uptake by ocular macrophages or retinal pigment epithelial cells that may harbor intracellular *M. tuberculosis* (Agnihotri et al., 2017; Baranyai et al., 2021) (See Figure 3: Advanced ocular delivery systems). Drug delivery and scaffold optimization are interdependent. Semi-synthetic derivatives of flavonoids with improved lipophilicity penetrate the cornea more effectively when delivered via nanoparticles or hydrogels (Gabai et al., 2023). Hybrid molecules combining two natural scaffolds can leverage synergistic antimicrobial activity while allowing incorporation into controlled-release delivery platforms (Yoon et al., 2025). Computational modeling can simulate drug distribution within ocular tissues, guiding scaffold selection and delivery design to maximize therapeutic outcomes (Kavousanakis et al., 2014; Pak et al., 2018).

**FIGURE 4:**
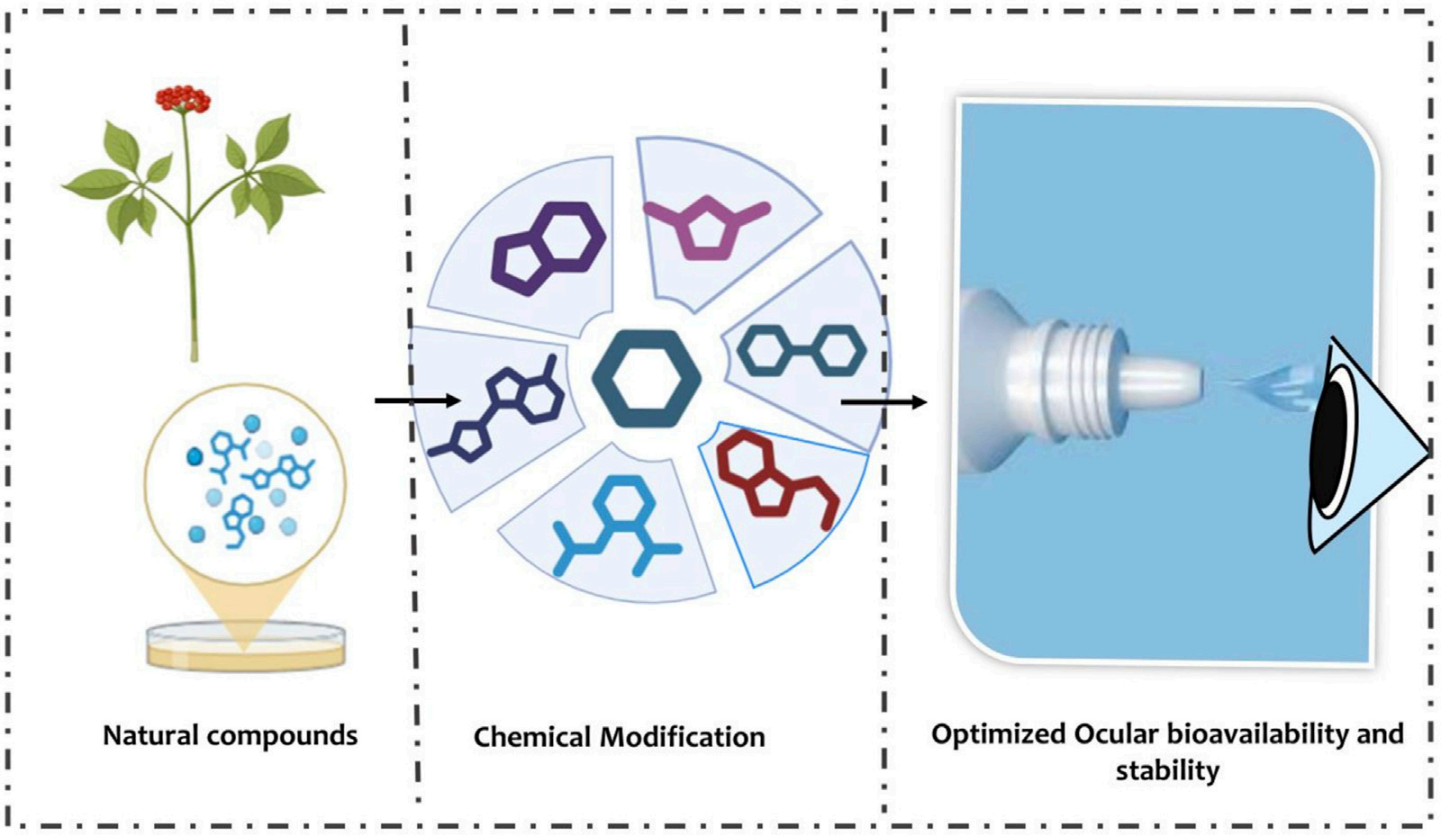
Scaffold Optimization and Synthetic Strategies. Flowchart schematic illustrating the progression from natural compounds through chemical modification approaches (prodrugs, esterification, hybrid molecules) to achieve optimized ocular bioavailability and stability. Arrows indicate enhanced corneal penetration, posterior segment delivery, and improved antimicrobial potency.

Advanced ocular drug delivery systems are therefore critical for translating the antimicrobial potential of natural compounds into effective therapies. By combining structural optimization, prodrug strategies, and innovative formulation platforms, sustained therapeutic concentrations can be achieved in both anterior and posterior segments, systemic toxicity can be minimized, and patient compliance can be improved. These approaches provide a pathway for the development of next-generation natural antibacterial ocular drugs capable of addressing the unmet clinical need in ocular tuberculosis as seen in Figure 4: Scaffold optimization and synthetic strategies for enhanced drug delivery in ocular TB.

## 5 Preclinical evaluation, clinical translation, and regulatory considerations

**FIGURE 5:**
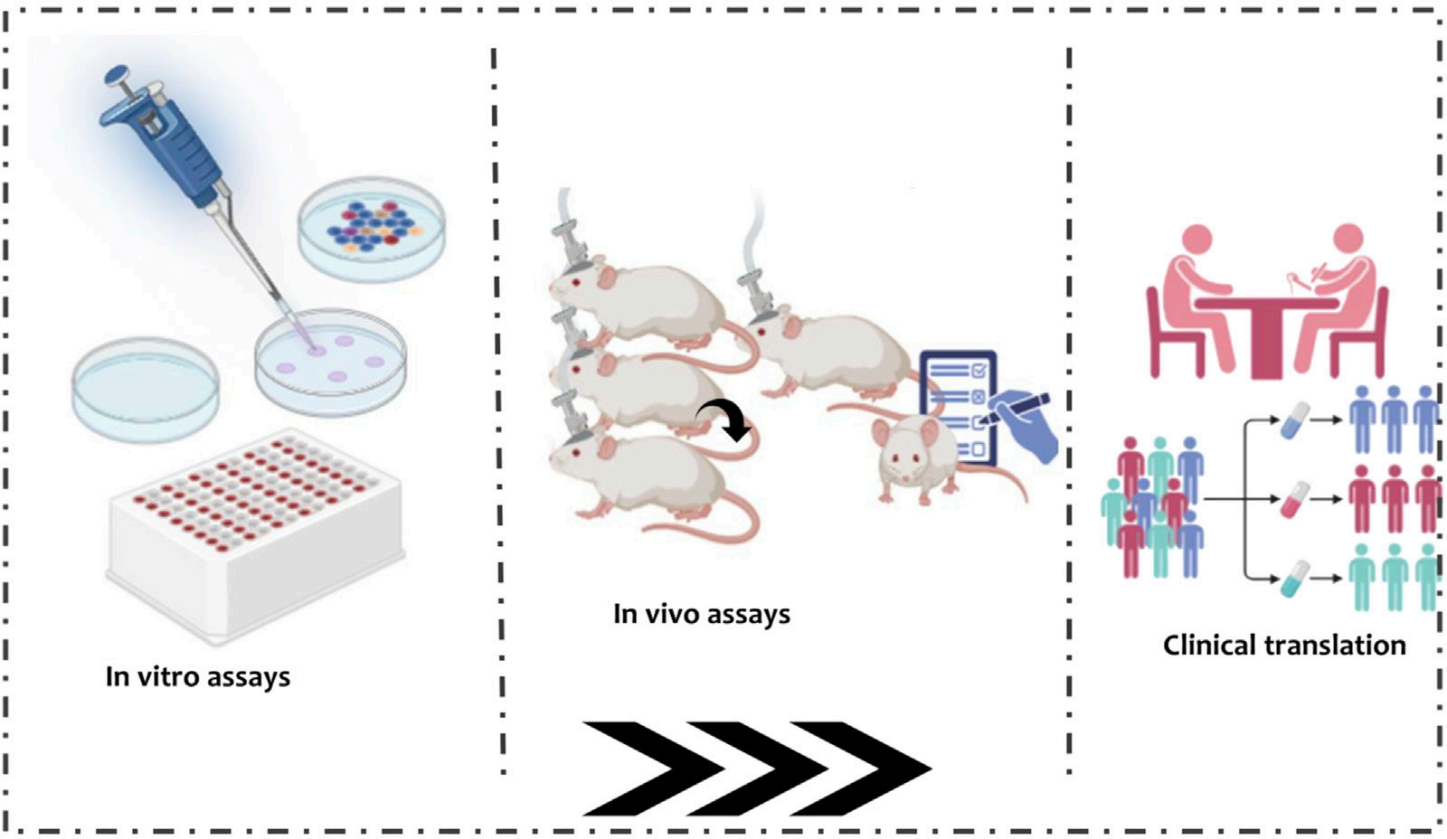
Preclinical evaluation pipeline. Stepwise schematic outlining the progression from in vitro assays (cell culture, MIC testing) through ex vivo ocular tissue diffusion studies and in vivo models (rabbit, guinea pig, non- human primate), culminating in clinical translation. Icons overlay pharmacokinetic distribution and tissue retention data to illustrate drug behavior across stages.

Preclinical studies are essential for establishing the safety, efficacy, and pharmacokinetic properties of natural antibacterial compounds intended for ocular tuberculosis therapy (Alffenaar et al., 2022) as seen in Figure 5: Preclinical evaluation pipeline.

Initial evaluation typically involves *in vitro* assays to determine minimum inhibitory concentrations and time-kill kinetics against *M. tuberculosis*, with surrogate organisms such as *Mycobacterium bovis* BCG often used for preliminary screening (Altaf, 2012; Arain et al., 1996). Cytotoxicity studies in ocular cell lines including human corneal epithelial cells, retinal pigment epithelial cells, and trabecular meshwork cells assess potential toxicity and guide compound selection (Ayaki et al., 2012). *Ex vivo* studies using excised ocular tissues provide insight into drug permeability and retention, with corneal, scleral, and retinal tissues mounted in diffusion chambers to measure compound flux, tissue binding, and metabolism (Begum et al., 2020; Kansara and Mitra, 2006). Techniques such as confocal microscopy and high-performance liquid chromatography allow precise quantification of compound distribution across ocular layers (Leung et al., 2024), which is particularly important for natural compounds due to their physicochemical diversity.

*In vivo* animal models offer critical evaluation of both efficacy and safety in a living system (Morgan et al., 2013). Rabbits are commonly employed because their ocular size and anatomy facilitate topical, intravitreal, and periocular administration (ZerniiE. et al., 2016). Guinea pigs and non-human primates provide additional immunocompetent models for systemic TB dissemination to ocular tissues (Flynn et al., 2015; Peña and Ho, 2017). Pharmacokinetic studies measure intraocular drug concentrations over time (Agrahari et al., 2016), while histopathology, fundus imaging, and optical coherence tomography assess tissue integrity and therapeutic response, with special attention to retinal and optic nerve safety given the risk of vision impairment (Moreira-Neto et al., 2019; Zimmerman et al., 2021).

Translating preclinical findings into human therapy requires careful planning. Diagnosis of ocular TB is often challenging because microbiological confirmation from ocular samples is difficult (Basu et al., 2014; Betzler et al., 2021); clinical studies therefore rely on systemic TB confirmation, imaging modalities, immunologic assays, and therapeutic response. Further, treatment endpoints typically include resolution of anterior uveitis, regression of choroidal granulomas, improvement of retinal vasculitis, and visual acuity outcomes (Agrawal et al., 2017; Elangovan et al., 2019). Natural antibacterial compounds offer potential advantages for clinical translation due to inherent biocompatibility and reduced systemic toxicity (Pancu et al., 2021). Their integration with systemic TB therapy requires consideration of drug–drug interactions, additive toxicity, and potential synergistic effects (Sieniawska et al., 2018). Combination therapy strategies may allow lower systemic doses while achieving high local ocular concentrations, enhancing both efficacy and safety.

Natural compounds also face unique regulatory challenges. Agencies such as the U.S. Food and Drug Administration and European Medicines Agency require comprehensive data on pharmacology, toxicology, and manufacturing quality (Goda, 2022). Demonstration of ocular safety and efficacy is paramount, particularly for compounds delivered via invasive routes such as intravitreal injections (Dzięgielewska et al., 2025). Stability, sterility, and reproducibility of natural product formulations must meet stringent standards (Hasegawa et al., 2021). Affordability and accessibility are additional considerations, particularly in high-burden, resource-limited countries where ocular TB is most prevalent (Foo et al., 2022). Scalability of compound extraction or synthesis, cost-effective formulation strategies, and cold-chain logistics for implants or nanoparticles are important determinants of successful deployment. Ethical considerations, including informed consent for invasive delivery methods and protection of vulnerable populations, must also be integrated into clinical trial design.

Preclinical and translational evaluation must further consider integration with advanced drug delivery systems. Nanoparticles, hydrogels, and sustained-release implants should be tested not only for pharmacokinetics but also for biocompatibility, inflammatory response, and long-term stability. Computational modeling and pharmacokinetic simulations can guide dosing strategies, optimize delivery routes, and predict tissue distribution, enhancing the efficiency of clinical translation. By combining rigorous preclinical assessment, careful clinical trial design, and adherence to regulatory standards, natural antibacterial compounds can be effectively developed into ocular TB therapies. Such integrated approaches promise to overcome historical barriers of drug penetration, toxicity, and compliance, providing safe and efficacious treatment options for patients worldwide.

## 6 Conclusion

In summary, Ocular TB remains a significant cause of preventable vision loss worldwide. Conventional systemic anti-TB therapies are limited by poor ocular penetration, prolonged treatment durations, and potential toxicity. Natural antibacterial compounds—including alkaloids, flavonoids, terpenoids, polyphenols, and saponins—offer a promising alternative due to their structural diversity, antimicrobial potency, and favorable biocompatibility. Advanced chemical optimization, such as prodrug strategies and scaffold modifications, combined with innovative ocular delivery systems like nanoparticles, hydrogels, and sustained-release implants, can overcome anatomical barriers to achieve therapeutic intraocular concentrations. Rigorous preclinical evaluation, careful clinical trial design, and adherence to regulatory standards are essential to translate these therapies into clinical practice. By integrating medicinal chemistry, formulation science, translational research, and global health strategies, next-generation natural antibacterial ocular drugs have the potential to prevent vision loss and improve quality of life for affected patients.

## 7 Future directions

The future of ocular TB therapy lies at the intersection of natural antibacterial compounds, modern drug design, advanced drug delivery systems, and personalized medicine. Computational modeling and artificial intelligence can accelerate the identification and optimization of natural scaffolds with high antimycobacterial potency, favorable ocular pharmacokinetics, and minimal toxicity. Structure-activity relationship analyses and molecular docking simulations can guide rational modifications to enhance corneal penetration, intraocular retention, and tissue specificity. Personalized therapy represents a particularly promising frontier. Advanced imaging modalities, such as optical coherence tomography (OCT) and fundus autofluorescence (FAF), can monitor lesion progression and guide individualized dosing schedules and drug selection. Pharmacogenomic profiling may predict systemic drug metabolism, transporter expression, and susceptibility to ocular toxicity, enabling patient-specific optimization. Combining these approaches with sustained-release nanoparticles, hydrogels, or implantable systems could reduce dosing frequency, improve adherence, and enhance therapeutic outcomes, particularly in resource-limited settings. Global health considerations are central to future progress. Affordable, scalable, and accessible therapies are essential in low- and middle-income countries where ocular TB prevalence is highest. Strategies such as simplified extraction, semi-synthetic modification of natural compounds, low-cost formulation platforms, and decentralized manufacturing can promote equitable access. Public-private partnerships and international research collaborations will be key to translating laboratory innovations into practical clinical solutions. Ethical oversight, particularly in vulnerable populations, remains critical to ensure safe and effective implementation. The convergence of natural product research and advanced ocular pharmacology offers a promising pathway toward safe, effective, and globally accessible therapies for ocular tuberculosis.

## Abbreviations

TB, Tuberculosis; Mtb, *M. tuberculosis*; ATT, Antitubercular Therapy; RPE, Retinal Pigment Epithelium; Nrf2, Nuclear factor erythroid 2-related factor 2; EGCG, Epigallocatechin gallate; NF-κB, Nuclear Factor kappa-light-chain-enhancer of activated B cells; IL, Interleukin; TNF-α, Tumor Necrosis Factor-alpha; MDR-TB, Multidrug-Resistant Tuberculosis; SAR, Structure–Activity Relationship; AI, Artificial Intelligence; PLGA, Poly(lactic-co-glycolic acid); HPLC, High-Performance Liquid Chromatography; OCT, Optical Coherence Tomography; FAF, Fundus Autofluorescence; PK, Pharmacokinetics; FDA, U.S. Food and Drug Administration; EMA, European Medicines Agency.

## References

[B1] Agarwal R. Iezhitsa I. Agarwal P. Abdul Nasir N. A. Razali N. Alyautdin R. (2016). Liposomes in topical ophthalmic drug delivery: an update. Drug Deliv. 23 (4), 1075–1091. 10.3109/10717544.2014.943336 25116511

[B2] Aggarwal R. Sethi P. Duvesh R. K. Sethi H. S. Naik M. Rai H. K. (2021). Ocular toxicity of anti-tubercular drugs. Delhi J. Ophthalmol. 31 (4), 35–38. 10.7869/djo.653

[B3] Agnihotri J. Singh S. Wais M. Pathak A. (2017). Macrophage targeted cellular carriers for effective delivery of anti-tubercular drugs. Recent Pat. Antiinfect Drug Discov. 12 (2), 162–183. 10.2174/1574891x13666171207151313 29219058

[B4] Agrahari V. Mandal A. Agrahari V. Trinh H. M. Joseph M. Ray A. (2016). A comprehensive insight on ocular pharmacokinetics. Drug Deliv. Transl. Res. 6 (6), 735–754. 10.1007/s13346-016-0339-2 27798766 PMC5319401

[B5] Agrawal R. Gunasekeran D. V. Grant R. Agarwal A. Kon O. M. Nguyen Q. D. (2017). Clinical features and outcomes of patients with tubercular uveitis treated with antitubercular therapy in the collaborative ocular tuberculosis study (COTS)–1. JAMA Ophthalmol. 135 (12), 1318–1327. 10.1001/jamaophthalmol.2017.4485 29075752 PMC6583556

[B6] Agrawal R. Gunasekeran D. V. Raje D. Agarwal A. Nguyen Q. D. Kon O. M. (2018). Global variations and challenges with tubercular uveitis in the collaborative ocular tuberculosis study. Invest Ophthalmol. Vis. Sci. 59 (10), 4162–4171. 10.1167/iovs.18-24102 30120485

[B7] Ahmad W. Arif M. M. Butt M. A. A. Manzoor A. Umer M. Naqvi S. Z. H. (2024). Anti-oxidant, anti-inflammatory, immunomodulatory and anti-pathogenic properties of Black seed (Nigella sativa) and its components, a review. J. Basic Emerg. Sci. 1 (2), 30–60.

[B8] Ahmed S. Amin M. M. Sayed S. (2023). Ocular drug delivery: a comprehensive review. AAPS PharmSciTech 24 (2), 66. 10.1208/s12249-023-02516-9 36788150

[B9] Akhter M. H. Ahmad I. Alshahrani M. Y. Al-Harbi A. I. Khalilullah H. Afzal O. (2022). Drug delivery challenges and current progress in nanocarrier-based ocular therapeutic system. Gels 8 (2), 82. 10.3390/gels8020082 35200463 PMC8871777

[B10] Akkerman O. W. Guenther G. Munoz-Torrico M. Babalik A. Heyckendorf J. Zellweger J.-P. (2023). Clinical presentation of pulmonary and extrapulmonary tuberculosis. Chall. Tuberc. 21st Century ERS Monogr. 101 (101)–51. 10.1183/2312508X.10005523

[B11] Akyakar B. Şahin İ. N. Ağagündüz D. Szép D. Budán F. (2025). The effect of curcumin on postmenopausal symptoms: a systematic review based on randomized controlled trials. Int. J. Mol. Sci. 26 (17), 8260. 10.3390/ijms26178260 40943181 PMC12428695

[B12] Albert D. M. Raven M. L. (2017). “Ocular tuberculosis,” in Tuberculosis and nontuberculous mycobacterial infections, 313–330.

[B13] Alffenaar J. C. de Steenwinkel J. E. M. Diacon A. H. Simonsson U. S. H. Srivastava S. Wicha S. G. (2022). Pharmacokinetics and pharmacodynamics of anti-tuberculosis drugs: an evaluation of *in vitro*, *in vivo* methodologies and human studies. Front. Pharmacol. 13, 1063453. 10.3389/fphar.2022.1063453 36569287 PMC9780293

[B14] Aljebreen M. A. Alotaibi A. K. Alrobaian M. (2020). Linezolid-induced toxic optic neuropathy. MIDDLE EAST Afr. J. Ophthalmol. 27 (4), 235–237. 10.4103/meajo.MEAJO_73_20 33814822 PMC7993054

[B15] Almeida H. Amaral M. H. Lobão P. Lobo J. M. S. (2014). *In situ* gelling systems: a strategy to improve the bioavailability of ophthalmic pharmaceutical formulations. Drug Discov. Today, 19(4), 400–412. 10.1016/j.drudis.2013.10.001 24120893

[B16] Altaf M. (2012). Evaluation of different mycobacterial species for drug discovery and characterisation of novel inhibitors of Mycobacterium tuberculosis

[B17] Anand U. Jacobo-Herrera N. Altemimi A. Lakhssassi N. (2019). A comprehensive review on medicinal plants as antimicrobial therapeutics: potential avenues of biocompatible drug discovery. Metabolites 9 (11), 258. 10.3390/metabo9110258 31683833 PMC6918160

[B18] Appiah-Opong R. Commandeur J. N. van Vugt-Lussenburg B. Vermeulen N. P. (2007). Inhibition of human recombinant cytochrome P450s by curcumin and curcumin decomposition products. Toxicology 235 (1-2), 83–91. 10.1016/j.tox.2007.03.007 17433521

[B19] Arabpour Z. Salehi M. An S. Moghtader A. Anwar K. N. Baharnoori S. M. (2024). Exploring hydrogel nanoparticle systems for enhanced ocular drug delivery. Gels 10 (9), 589. 10.3390/gels10090589 39330191 PMC11430953

[B20] Arain T. M. Resconi A. E. Hickey M. J. Stover C. K. (1996). Bioluminescence screening *in vitro* (Bio-Siv) assays for high-volume antimycobacterial drug discovery. Antimicrob. Agents Chemother. 40 (6), 1536–1541. 10.1128/aac.40.6.1536 8726034 PMC163364

[B21] Ashrafizadeh M. Ahmadi Z. Mohammadinejad R. Farkhondeh T. Samarghandian S. (2020). Curcumin activates the Nrf2 pathway and induces cellular protection against oxidative injury. Curr. Mol. Med. 20 (2), 116–133. 10.2174/1566524019666191016150757 31622191

[B22] Ayaki M. Iwasawa A. Niwano Y. (2012). Comparative assessment of the cytotoxicity of six anti-inflammatory eyedrops in four cultured ocular surface cell lines, as determined by cell viability scores. Clin. Ophthalmol. 6, 1879–1884. 10.2147/OPTH.S36968 23185116 PMC3501841

[B23] Aydin E. Gunduz M. K. Kaymak G. Sezgin A. K. Dağgez H. Renders D. P. (2025). Antimycobacterial activity of luteolin in resistant *Mycobacterium tuberculosis* isolates and cytotoxicity on L929 cells. Microb. Pathog., 200, 107287. 10.1016/j.micpath.2025.107287 39800168

[B24] Badary O. A. Al-Shabanah O. A. Nagi M. N. Al-Bekairi A. M. Elmazar M. M. A. (1998). Acute and subchronic toxicity of thymoquinone in mice. Drug Dev. Res., 44(2-3), 56–61. 10.1002/(SICI)1098-2299(199806/07)44:2/3<56::AID-DDR2>3.0.CO;2-9

[B25] Bagcchi S. (2023). WHO's global Tuberculosis report 2022. Lancet Microbe 4 (1), e20. 10.1016/S2666-5247(22)00359-7 36521512

[B26] Baptista-Silva S. Borges S. Ramos O. L. Pintado M. Sarmento B. (2020). The progress of essential oils as potential therapeutic agents: a review. J. Essent. Oil Res. 32 (4), 279–295. 10.1080/10412905.2020.1746698

[B27] Baranyai Z. Soria-Carrera H. Alleva M. Millán-Placer A. C. Lucía A. Martín-Rapún R. (2021). Nanotechnology-based targeted drug delivery: an emerging tool to overcome tuberculosis. Adv. Ther., 4(1), 2000113. 10.1002/adtp.202000113

[B28] Bashiri G. Nigon L. V. Jirgis E. N. Ho N. A. T. Stanborough T. Dawes S. S. (2020). Allosteric regulation of menaquinone (vitamin K2) biosynthesis in the human pathogen *Mycobacterium tuberculosis* . J. Biol. Chem. 295 (12), 3759–3770. 10.1074/jbc.RA119.012158 32029475 PMC7086027

[B29] Basu S. (2022). Absence of evidence as the evidence of absence: the curious case of latent infection causing ocular tuberculosis. Front. Ophthalmol. 2, 874400. 10.3389/fopht.2022.874400 35911853 PMC7613174

[B30] Basu S. Monira S. Modi R. R. Choudhury N. Mohan N. Padhi T. R. (2014). Degree, duration, and causes of visual impairment in eyes affected with ocular tuberculosis. J. Ophthalmic Inflamm. Infect. 4 (1), 3. 10.1186/1869-5760-4-3 24485195 PMC3912920

[B31] Basu S. Wakefield D. Biswas J. Rao N. A. (2015). Pathogenesis and pathology of intraocular tuberculosis. Ocular Immunol. Inflamm. 23 (4), 353–357. 10.3109/09273948.2015.1056536 29265968

[B32] Basu S. Elkington P. Rao N. A. (2020). Pathogenesis of ocular tuberculosis: new observations and future directions. Tuberculosis, 124, 101961. 10.1016/j.tube.2020.101961 33010848

[B33] Begum G. Leigh T. Courtie E. Moakes R. Butt G. Ahmed Z. (2020). Rapid assessment of ocular drug delivery in a novel *ex vivo* corneal model. Sci. Rep. 10 (1), 11754. 10.1038/s41598-020-68254-1 32678110 PMC7366725

[B34] Betzler B. K. Gupta V. Agrawal R. (2021). Clinics of ocular tuberculosis: a review. Clin. and Exp. Ophthalmol., 49(2), 146–160. 10.1111/ceo.13847 33429468

[B35] Betzler B. K. Putera I. Testi I. La Distia Nora R. Kempen J. Kon O. M. (2023). Anti-tubercular therapy in the treatment of tubercular uveitis: a systematic review and meta-analysis. Surv. Ophthalmol., 68(2), 241–256. 10.1016/j.survophthal.2022.10.001 36272559

[B36] Bhambhani S. Kondhare K. R. Giri A. P. (2021). Diversity in chemical structures and biological properties of plant alkaloids. Molecules 26 (11), 3374. 10.3390/molecules26113374 34204857 PMC8199754

[B37] Bhandari M. Nguyen S. Yazdani M. Utheim T. P. Hagesaether E. (2022). The therapeutic benefits of nanoencapsulation in drug delivery to the anterior segment of the eye: a systematic review. Front. Pharmacol. 13, 903519. 10.3389/fphar.2022.903519 35645827 PMC9136980

[B38] Bhat Z. S. Rather M. A. Maqbool M. Ahmad Z. (2018). Drug targets exploited in mycobacterium tuberculosis: pitfalls and promises on the horizon. Biomed. and Pharmacother., 103, 1733–1747. 10.1016/j.biopha.2018.04.176 29864964

[B39] Bhia M. Motallebi M. Abadi B. Zarepour A. Pereira-Silva M. Saremnejad F. (2021). Naringenin nano-delivery systems and their therapeutic applications. Pharmaceutics 13 (2), 291. 10.3390/pharmaceutics13020291 33672366 PMC7926828

[B40] Bishnoi P. Saroha B. Kumar S. Kumar G. Bhardwaj A. Kumari M. (2025). Click chemistry: an overview and recent updates in the medicinal attributes of click-derived heterocycles. Mol. Divers. 10.1007/s11030-025-11110-z 39918713

[B41] Biswas A. Kumar S. Choudhury A. D. Bisen A. C. Sanap S. N. Agrawal S. (2024). Polymers and their engineered analogues for ocular drug delivery: enhancing therapeutic precision. Biopolymers 115 (4), e23578. 10.1002/bip.23578 38577865

[B42] Breijyeh Z. Karaman R. (2023). Design and synthesis of novel antimicrobial agents. Antibiotics 12 (3), 628. 10.3390/antibiotics12030628 36978495 PMC10045396

[B43] Brindha P. (2016). Role of phytochemicals as immunomodulatory agents: a review. Int. J. Green Pharm. (IJGP) 10 (1). 10.22377/ijgp.v10i1.600

[B44] Caban M. Owczarek K. Chojnacka K. Lewandowska U. (2022). Overview of polyphenols and polyphenol-rich extracts as modulators of inflammatory response in dry eye syndrome. Food Rev. Int. 38 (Suppl. 1), 501–528. 10.1080/87559129.2021.1874412

[B45] Cabrera F. J. Wang D. C. Reddy K. Acharya G. Shin C. S. (2019). Challenges and opportunities for drug delivery to the posterior of the eye. Drug Discov. Today, 24(8), 1679–1684. 10.1016/j.drudis.2019.05.035 31175955 PMC6708448

[B46] Cai Y. Ma W. Xiao Y. Wu B. Li X. Liu F. (2017). High doses of baicalin induces kidney injury and fibrosis through regulating TGF-β/Smad signaling pathway. Toxicol. Appl. Pharmacol. 333, 1–9. 10.1016/j.taap.2017.08.003 28803990

[B47] Cetin M. (2021). A review on ophthalmic delivery systems containing flavonoids for the treatment of eye diseases. NanoEra 1 (1), 1–13.

[B48] Chadar D. Camilles M. Patil R. Khan A. Weyhermüller T. Salunke-Gawali S. (2015). Synthesis and characterization of n-alkylamino derivatives of vitamin K3: molecular structure of 2-propylamino-3-methyl-1, 4-naphthoquinone and antibacterial activities. J. Mol. Struct. 1086, 179–189. 10.1016/j.molstruc.2015.01.029

[B49] Chen Y. Xiao C.-Q. He Y.-J. Chen B.-L. Wang G. Zhou G. (2011). Genistein alters caffeine exposure in healthy female volunteers. Eur. J. Clin. Pharmacol. 67 (4), 347–353. 10.1007/s00228-010-0964-5 21222115

[B50] Chen J. Jiang Q.-D. Chai Y.-P. Zhang H. Peng P. Yang X.-X. (2016). Natural terpenes as penetration enhancers for transdermal drug delivery. Molecules 21 (12), 1709. 10.3390/molecules21121709 27973428 PMC6273457

[B51] Cholkar K. Dasari S. R. Pal D. Mitra A. K. (2013). Eye: anatomy, physiology and barriers to drug delivery. Ocular Transporters and Receptors, 1–36. Woodhead Publishing. 10.1533/9781908818317.1

[B52] de Oliveira N. K. Almeida M. R. S. Pontes F. M. M. Barcelos M. P. Silva G. M. de Paula da Silva C. H. T. (2021). Molecular docking, physicochemical properties, pharmacokinetics and toxicity of flavonoids present in Euterpe oleracea martius. Curr. Computer-Aided Drug Des. 17 (4), 589–617. 10.2174/1573409916666200619122803 32560610

[B53] Delmas D. Cornebise C. Courtaut F. Xiao J. Aires V. (2021). New highlights of resveratrol: a review of properties against ocular diseases. Int. J. Mol. Sci. 22 (3), 1295. 10.3390/ijms22031295 33525499 PMC7865717

[B54] Desai N. Colley H. E. Krishna Y. Bosworth L. A. Kearns V. R. (2025). Mucoadhesive nanofibers for ocular drug delivery: mechanisms, design strategies, and applications. Drug Deliv. Transl. Res. 10.1007/s13346-025-01894-w 40562965 PMC12956969

[B55] Dzięgielewska M. Tomczyk M. Wiater A. Woytoń A. Junka A. (2025). Targeting ocular biofilms with plant-derived antimicrobials in the era of antibiotic resistance. Molecules 30 (13), 2863. 10.3390/molecules30132863 40649377 PMC12251026

[B56] Elangovan S. Govindarajan S. Mayilvakanam L. Gunasekaran N. (2019). Clinical profile and treatment response of patients with ocular inflammation due to presumed ocular tuberculosis: a retrospective study. Turkish J. Ophthalmol. 49 (4), 188–193. 10.4274/tjo.galenos.2019.05874 31486605 PMC6761386

[B57] Farid R. M. El-Salamouni N. S. El-Kamel A. H. El-Gamal S. S. (2017). Chapter 16 - lipid-based nanocarriers for ocular drug delivery. In Andronescu E. Grumezescu A. M. (Eds.), Nanostructures for drug delivery (pp. 495–522). Elsevier. 10.1016/B978-0-323-46143-6.00016-6

[B58] Farzan M. Farzan M. Shahrani M. Navabi S. P. Vardanjani H. R. Amini-Khoei H. (2024). Neuroprotective properties of betulin, betulinic acid, and Ursolic acid as triterpenoids derivatives: a comprehensive review of mechanistic studies. Nutr. Neurosci. 27 (3), 223–240. 10.1080/1028415X.2023.2180865 36821092

[B59] Flynn J. L. Gideon H. P. Mattila J. T. Lin P. L. (2015). Immunology studies in non-human primate models of tuberculosis. Immunol. Rev., 264(1), 60–73. 10.1111/imr.12258 25703552 PMC4339213

[B60] Foo C. D. Shrestha P. Wang L. Du Q. García-Basteiro A. L. Abdullah A. S. (2022). Integrating tuberculosis and noncommunicable diseases care in low- and middle-income countries (LMICs): a systematic review. PLOS Med. 19 (1), e1003899. 10.1371/journal.pmed.1003899 35041654 PMC8806070

[B61] Francis G. Kerem Z. Makkar H. P. Becker K. (2002). The biological action of saponins in animal systems: a review. Br. J. Nutr. 88 (6), 587–605. 10.1079/BJN2002725 12493081

[B62] Franzone F. Nebbioso M. Pergolizzi T. Attanasio G. Musacchio A. Greco A. (2021). Anti-inflammatory role of curcumin in retinal disorders (review). Exp. Ther. Med. 22 (1), 790. 10.3892/etm.2021.10222 34055089 PMC8145690

[B63] Gabai A. Zeppieri M. Finocchio L. Salati C. (2023). Innovative strategies for drug delivery to the ocular posterior segment. Pharmaceutics 15 (7), 1862. 10.3390/pharmaceutics15071862 37514050 PMC10385847

[B64] Gajbhiye S. Pal K. (2024). Toxic and allergic responses caused by secondary metabolites used in cosmetic formulations. 73, 104. 10.1039/9781837672288-00073

[B65] Gallegos M. T. Vargas Gallego P. A. Rodríguez-García I. (2016). Antibacterial actions of flavonoids.

[B66] Ganapathy U. S. Lan T. Krastel P. Lindman M. Zimmerman M. D. Ho H. (2021). A *Mycobacterium tuberculosis* NBTI DNA gyrase inhibitor is active against Mycobacterium abscessus. Antimicrob. Agents Chemother. 65 (9), e0151421. 10.1128/AAC.01514-21 34606340 PMC8597734

[B67] Gangwal A. Lavecchia A. (2025). Artificial intelligence in natural product drug discovery: current applications and future perspectives. J. Med. Chem. 68 (4), 3948–3969. 10.1021/acs.jmedchem.4c01257 39916476 PMC11874025

[B68] Gegia M. Winters N. Benedetti A. van Soolingen D. Menzies D. (2017). Treatment of isoniazid-resistant tuberculosis with first-line drugs: a systematic review and meta-analysis. Lancet Infect. Dis. 17 (2), 223–234. 10.1016/S1473-3099(16)30407-8 27865891

[B69] Ghosh D. Yadav S. Bag S. Mallick A. I. De P. (2024). Antibacterial activity of hydrophobicity modulated cationic polymers with enzyme and pH-responsiveness. J. Mater. Chem. B 12 (11), 2894–2904. 10.1039/d3tb02801a 38436419

[B70] Gilani S. J. Jumah M. N. b. Zafar A. Imam S. S. Yasir M. Khalid M. (2022). Formulation and evaluation of nano lipid carrier-based ocular gel system: optimization to antibacterial activity. Gels 8 (5), 255. 10.3390/gels8050255 35621552 PMC9140781

[B71] Goda Y. (2022). Regulatory science of natural products. J. Nat. Med. 76 (4), 732–747. 10.1007/s11418-022-01639-w 35870047 PMC9307968

[B72] Guelfi G. Pasquariello R. Anipchenko P. Capaccia C. Pennarossa G. Brevini T. A. L. (2023). The role of genistein in mammalian reproduction. Molecules 28 (21), 7436. 10.3390/molecules28217436 37959856 PMC10647478

[B73] Gunda S. Hariharan S. Mandava N. Mitra A. K. (2008). “Barriers in ocular drug delivery,” in Ocular transporters in ophthalmic diseases and drug delivery: ophthalmology research (Springer), 399–413.

[B74] Guo Y. Chen Y. Tan Z.-r. Klaassen C. D. Zhou H.-h. (2012). Repeated administration of berberine inhibits cytochromes P450 in humans. Eur. J. Clin. Pharmacol. 68 (2), 213–217. 10.1007/s00228-011-1108-2 21870106 PMC4898966

[B75] Gupta V. Gupta A. Rao N. A. (2007). Intraocular tuberculosis—an update. Surv. Ophthalmol., 52(6), 561–587. 10.1016/j.survophthal.2007.08.015 18029267

[B76] Gupta V. Bansal R. Gupta A. (2011). Continuous progression of tubercular serpiginous-like choroiditis after initiating antituberculosis treatment. Am. J. Ophthalmol., 152(5), 857–863. 10.1016/j.ajo.2011.05.004 21794847

[B77] Gupta A. Sharma A. Bansal R. Sharma K. (2015). Classification of intraocular tuberculosis. Ocular Immunol. Inflamm. 23 (1), 7–13. 10.3109/09273948.2014.967358 25314361

[B78] Gupta V. Shoughy S. S. Mahajan S. Khairallah M. Rosenbaum J. T. Curi A. (2015). Clinics of ocular tuberculosis. Ocular Immunol. Inflamm. 23 (1), 14–24. 10.3109/09273948.2014.986582 25615807

[B79] Gupta B. Mishra V. Gharat S. Momin M. Omri A. (2021). Cellulosic polymers for enhancing drug bioavailability in ocular drug delivery systems. Pharmaceuticals 14 (11), 1201. 10.3390/ph14111201 34832983 PMC8621906

[B80] Halicki P. C. Ferreira L. A. De Moura K. C. Carneiro P. F. Del Rio K. P. Carvalho T. d. S. (2018). Naphthoquinone derivatives as scaffold to develop new drugs for tuberculosis treatment. Front. Microbiol. 9, 673. 10.3389/fmicb.2018.00673 29686657 PMC5900025

[B81] Hannan M. A. Rahman M. A. Sohag A. A. M. Uddin M. J. Dash R. Sikder M. H. (2021). Black cumin (Nigella sativa L.): a comprehensive review on phytochemistry, health benefits, molecular pharmacology, and safety. Nutrients 13 (6), 1784. 10.3390/nu13061784 34073784 PMC8225153

[B82] Hasanuzzaman M. Yi M. Cho M. Parvez M. M. Lee S.-J. Shin J.-G. (2019). Rifampin induces expression of P-glycoprotein on the THP1 cell–derived macrophages, causing decrease intramacrophage concentration of prothionamide. J. Pharm. Sci. 108 (9), 3106–3111. 10.1016/j.xphs.2019.04.009 30991038

[B83] Hasegawa A. Gulmezian-Sefer M. Cheng Y. Srikumar R. (2021). “Microbiological considerations for ophthalmic products: sterility, endotoxin limits, and preservatives,” in Ophthalmic product development: from bench to bedside. Editors Neervannan S. Kompella U. B. (Springer International Publishing), 199–227. 10.1007/978-3-030-76367-1_9

[B84] Hewlings S. J. Kalman D. S. (2017). Curcumin: a review of its effects on human health. Foods 6 (10), 92. 10.3390/foods6100092 29065496 PMC5664031

[B85] Hu Y. Cheng Z. Heller L. I. Krasnoff S. B. Glahn R. P. Welch R. M. (2006). Kaempferol in red and pinto bean seed (Phaseolus vulgaris L.) coats inhibits iron bioavailability using an *in vitro* digestion/human Caco-2 cell model. J. Agric. Food Chem. 54 (24), 9254–9261. 10.1021/jf0612981 17117818

[B86] Hu X. Liang Y. Zhao B. Wang Y. (2019). Thymoquinone protects human retinal pigment epithelial cells against hydrogen peroxide induced oxidative stress and apoptosis. J. Cell. Biochem. 120 (3), 4514–4522. 10.1002/jcb.27739 30269355

[B87] Hytti M. Piippo N. Korhonen E. Honkakoski P. Kaarniranta K. Kauppinen A. (2015). Fisetin and luteolin protect human retinal pigment epithelial cells from oxidative stress-induced cell death and regulate inflammation. Sci. Rep. 5 (1), 17645. 10.1038/srep17645 26619957 PMC4664957

[B88] Imenshahidi M. Hosseinzadeh H. (2019). Berberine and barberry (berberis vulgaris): a clinical review. Phytotherapy Res., 33(3), 504–523. 10.1002/ptr.6252 30637820

[B89] Jacob S. Nair A. B. Shah J. Gupta S. Boddu S. H. Sreeharsha N. (2022). Lipid nanoparticles as a promising drug delivery carrier for topical ocular Therapy—An overview on recent advances. Pharmaceutics 14 (3), 533. 10.3390/pharmaceutics14030533 35335909 PMC8955373

[B90] Jagatap V. R. Ahmad I. Patel H. M. (2022). Recent updates in natural terpenoids as potential anti-mycobacterial agents. Indian J. Tuberc. 69 (3), 282–304. 10.1016/j.ijtb.2021.07.006 35760478

[B91] Jayaraman P. Sakharkar K. R. Lim C. Siddiqi M. I. Dhillon S. K. Sakharkar M. K. (2013). Novel phytochemical–antibiotic conjugates as multitarget inhibitors of pseudomononas aeruginosa GyrB/ParE and DHFR. Drug Des. Dev. Ther. 7 (null), 449–475. 10.2147/DDDT.S43964 23818757 PMC3692347

[B92] Jiménez-Arellanes A. Luna-Herrera J. Cornejo-Garrido J. López-García S. Castro-Mussot M. E. Meckes-Fischer M. (2013). Ursolic and oleanolic acids as antimicrobial and immunomodulatory compounds for tuberculosis treatment. BMC Complementary Altern. Med. 13 (1), 258. 10.1186/1472-6882-13-258 24098949 PMC3853017

[B93] Kaabi Y. A. (2022). Potential roles of anti-inflammatory plant-derived bioactive compounds targeting inflammation in microvascular complications of diabetes. Molecules 27 (21), 7352. 10.3390/molecules27217352 36364178 PMC9657994

[B94] Kansara V. Mitra A. K. (2006). Evaluation of an *Ex Vivo* model implication for carrier-mediated retinal drug delivery. Curr. Eye Res. 31 (5), 415–426. 10.1080/02713680600646890 16714233

[B95] Karnan A. Jadhav U. Ghewade B. Ledwani A. Shivashankar P. Jadhav U. (2024). A comprehensive review on long vs. short regimens in multidrug-resistant tuberculosis (MDR-TB) under programmatic management of drug-resistant tuberculosis (PMDT). Cureus 16 (1). 10.7759/cureus.52706 38384625 PMC10879947

[B96] Kavousanakis M. E. Kalogeropoulos N. G. Hatziavramidis D. T. (2014). Computational modeling of drug delivery to the posterior eye. Chem. Eng. Sci., 108, 203–212. 10.1016/j.ces.2014.01.005

[B97] Kee A. R. Gonzalez-Lopez J. J. Al-Hity A. Gupta B. Lee C. S. Gunasekeran D. V. (2016). Anti-tubercular therapy for intraocular tuberculosis: a systematic review and meta-analysis. Surv. Ophthalmol., 61(5), 628–653. 10.1016/j.survophthal.2016.03.001 26970263 PMC5061337

[B98] Keihanian F. Saeidinia A. Bagheri R. K. Johnston T. P. Sahebkar A. (2018). Curcumin, hemostasis, thrombosis, and coagulation. J. Cell. Physiology, 233(6), 4497–4511. 10.1002/jcp.26249 29052850

[B99] Koirala N. Modi B. Subba R. K. Panthi M. Xiao J. (2021). “Medicinal plants in targeting tuberculosis II,” in Medicinal plants for lung diseases: a pharmacological and immunological perspective. Editors Dua K. Nammi S. Chang D. Chellappan D. K. Gupta G. Collet T. (Singapore: Springer), 185–215. 10.1007/978-981-33-6850-7_8

[B100] Krstić L. González-García M. J. Diebold Y. (2021). Ocular delivery of polyphenols: meeting the unmet needs. Molecules 26 (2), 370. 10.3390/molecules26020370 33445725 PMC7828190

[B101] Kumar S. V. Saravanan D. Kumar B. Jayakumar A. (2014). An update on prodrugs from natural products. Asian Pac. J. Trop. Med., 7, S54–S59. 10.1016/S1995-7645(14)60203-0 25312180

[B102] Kumar P. Sulakhiya K. Barua C. C. Mundhe N. (2017). TNF-α, IL-6 and IL-10 expressions, responsible for disparity in action of curcumin against cisplatin-induced nephrotoxicity in rats. Mol. Cell. Biochem. 431 (1), 113–122. 10.1007/s11010-017-2981-5 28258441

[B103] Kumar M. Singh S. K. Singh P. P. Singh V. K. Rai A. C. Srivastava A. K. (2021). Potential anti-mycobacterium tuberculosis activity of plant secondary metabolites: insight with molecular docking interactions. Antioxidants 10 (12), 1990. 10.3390/antiox10121990 34943093 PMC8750514

[B104] Lakhanpal P. Rai D. K. (2007). Quercetin: a versatile flavonoid. Internet J. Med. Update 2 (2), 22–37. 10.4314/ijmu.v2i2.39851

[B105] Lançon A. Frazzi R. Latruffe N. (2016). Anti-oxidant, anti-inflammatory and anti-angiogenic properties of resveratrol in ocular diseases. Molecules 21 (3), 304. 10.3390/molecules21030304 26950104 PMC6272926

[B106] Lasoń E. (2020). Topical administration of terpenes encapsulated in nanostructured lipid-based systems. Molecules 25 (23), 5758. 10.3390/molecules25235758 33297317 PMC7730254

[B107] Leclercq I. A. Farrell G. C. Sempoux C. dela Peña A. Horsmans Y. (2004). Curcumin inhibits NF-kappaB activation and reduces the severity of experimental steatohepatitis in mice. J. hepatology 41 (6), 926–934. 10.1016/j.jhep.2004.08.010 15582125

[B108] Lee S. H. Kim H. Y. Back S. Y. Han H.-K. (2018). Piperine-mediated drug interactions and formulation strategy for piperine: recent advances and future perspectives. Expert Opin. Drug Metabolism and Toxicol. 14 (1), 43–57. 10.1080/17425255.2018.1418854 29250980

[B109] Leung M. Radhakrishnan R. Lor A. Li D. Yochim D. More S. (2024). Quantitative analysis of dietary vitamin A metabolites in murine ocular and non-ocular tissues using high-performance liquid chromatography. J. Vis. Exp. 214. 10.3791/67034 39803967

[B110] Li M. Xin M. Guo C. Lin G. Wu X. (2017). New nanomicelle curcumin formulation for ocular delivery: improved stability, solubility, and ocular anti-inflammatory treatment. Drug Dev. Ind. Pharm. 43 (11), 1846–1857. 10.1080/03639045.2017.1349787 28665151

[B111] Li D. L. Mao L. Gu Q. Wei F. Gong Y.-Y. (2021). Quercetin protects retina external barrier from oxidative stress injury by promoting autophagy. Cutan. Ocular Toxicol. 40 (1), 7–13. 10.1080/15569527.2020.1860082 33283549

[B112] Li J. Cui P. Jing H. Chen S. Ma L. Zhang W. (2024). Hydrogen combined with tetrandrine attenuates silica-induced pulmonary fibrosis *via* suppressing NF-kappaB/NLRP3 signaling pathway-mediated epithelial mesenchymal transition and inflammation. Int. Immunopharmacol. 138, 112563. 10.1016/j.intimp.2024.112563 38943976

[B113] Libershteyn Y. (2016). Ethambutol/linezolid toxic optic neuropathy. Optometry Vis. Sci. 93 (2), 211–217. 10.1097/OPX.0000000000000783 26636399

[B114] Liu R. Sun L. Fang S. Wang S. Chen J. Xiao X. (2016). Thermosensitive *in situ* nanogel as ophthalmic delivery system of curcumin: development, characterization, *in vitro* permeation and *in vivo* pharmacokinetic studies. Pharm. Dev. Technol. 21 (5), 576–582. 10.3109/10837450.2015.1026607 26024239

[B115] Liu Z. Li Y. Wen Y. Bao J. Tian L. Jie Y. (2025). Curcumin ameliorates benzalkonium chloride-induced dry eye disease in mice. Exp. Eye Res. 259, 110509. 10.1016/j.exer.2025.110509 40633646

[B116] López-Cano J. J. González-Cela-Casamayor M. A. Andrés-Guerrero V. Herrero-Vanrell R. Molina-Martínez I. T. (2021). Liposomes as vehicles for topical ophthalmic drug delivery and ocular surface protection. Expert Opin. drug Deliv. 18 (7), 819–847. 10.1080/17425247.2021.1872542 33412914

[B117] Lu H.-e. Chen Y. Sun X.-B. Tong B. Fan X.-H. (2015). Effects of luteolin on retinal oxidative stress and inflammation in diabetes. Rsc Adv. 5 (7), 4898–4904. 10.1039/c4ra10756j

[B118] Madge S. N. Prabhakaran V. C. Shome D. Kim U. Honavar S. Selva D. (2008). Orbital tuberculosis: a review of the literature. Orbit 27 (4), 267–277. 10.1080/01676830802225152 18716964

[B119] Mahmud N. M. Paraoan L. Khaliddin N. Kamalden T. A. (2022). Thymoquinone in ocular neurodegeneration: modulation of pathological mechanisms *via* multiple pathways. Front. Cell. Neurosci. 16, 786926. 10.3389/fncel.2022.786926 35308121 PMC8924063

[B120] Maiolini M. Gause S. Taylor J. Steakin T. Shipp G. Lamichhane P. (2020). The war against tuberculosis: a review of natural compounds and their derivatives. Molecules 25 (13), 3011. 10.3390/molecules25133011 32630150 PMC7412169

[B121] Mallik S. Mohanty J. N. Sahoo S. J. (2025). Active phytoconstituents to tackle the resistance mechanism of MDR and XDR bacteria: a critical review. Microbes Infect. Dis. 6 (3), 0–4019. 10.21608/mid.2024.297687.2004

[B122] Man-Ren R. (2002). Effects of tetrandrine on cardiac and vascular remodeling. Acta Pharmacol. Sin. 23 (12), 1075–1085. 12466044

[B123] Mandal M. N. A. Patlolla J. M. Zheng L. Agbaga M.-P. Tran J.-T. A. Wicker L. (2009). Curcumin protects retinal cells from light-and oxidant stress-induced cell death. Free Radic. Biol. Med. 46 (5), 672–679. 10.1016/j.freeradbiomed.2008.12.006 19121385 PMC2810836

[B124] Marrelli M. Conforti F. Araniti F. Statti G. A. (2016). Effects of saponins on lipid metabolism: a review of potential health benefits in the treatment of obesity. Molecules 21 (10), 1404. 10.3390/molecules21101404 27775618 PMC6273086

[B125] Martin D. A. Bolling B. W. (2015). A review of the efficacy of dietary polyphenols in experimental models of inflammatory bowel diseases. Food and Funct. 6 (6), 1773–1786. 10.1039/c5fo00202h 25986932

[B126] Mazlun M. H. Sabran S. F. Mohamed M. Abu Bakar M. F. Abdullah Z. (2019). Phenolic compounds as promising drug candidates in tuberculosis therapy. Molecules 24 (13), 2449. 10.3390/molecules24132449 31277371 PMC6651284

[B127] Mehjabin S. Akanda M. K. M. Hoque N. Hasan A. N. Parvez G. M. Mosaddik A. (2024). Flavonoid intake and risk of toxicity in chronic metabolic disease. *Role of flavonoids in chronic Metabolic diseases: from bench to clinic* , 511–534.

[B128] Memarzia A. Khazdair M. R. Behrouz S. Gholamnezhad Z. Jafarnezhad M. Saadat S. (2021). Experimental and clinical reports on anti‐inflammatory, antioxidant, and immunomodulatory effects of Curcuma longa and curcumin, an updated and comprehensive review. BioFactors 47 (3), 311–350. 10.1002/biof.1716 33606322

[B129] Mickymaray S. Alfaiz F. A. Paramasivam A. (2020). Efficacy and mechanisms of flavonoids against the emerging opportunistic nontuberculous mycobacteria. Antibiotics 9 (8), 450. 10.3390/antibiotics9080450 32726972 PMC7460331

[B130] Milman N. T. (2020). A review of nutrients and compounds, which promote or inhibit intestinal iron absorption: making a platform for dietary measures that can reduce iron uptake in patients with genetic haemochromatosis. J. Nutr. Metabolism 2020 (1), 7373498. 10.1155/2020/7373498 33005455 PMC7509542

[B131] Mishra G. P. Bagui M. Tamboli V. Mitra A. K. (2011). Recent applications of liposomes in ophthalmic drug delivery. J. Drug Deliv., 2011(1), 863734. 10.1155/2011/863734 21490757 PMC3066533

[B132] Mishra V. Soren A. D. Yadav A. K. (2021). Toxicological evaluations of betulinic acid and ursolic acid; common constituents of Houttuynia cordata used as an anthelmintic by the naga tribes in North-East India. Future J. Pharm. Sci. 7 (1), 39. 10.1186/s43094-020-00173-4

[B133] Mollazadeh H. Cicero A. F. Blesso C. N. Pirro M. Majeed M. Sahebkar A. (2019). Immune modulation by curcumin: the role of interleukin-10. Crit. Rev. food Sci. Nutr. 59 (1), 89–101. 10.1080/10408398.2017.1358139 28799796

[B134] Moreira E. S. Ames-Sibin A. P. Bonetti C. I. Leal L. E. Peralta R. M. de Sá-Nakanishi A. B. (2022). The short-term effects of berberine in the liver: narrow margins between benefits and toxicity. Toxicol. Lett. 368, 56–65. 10.1016/j.toxlet.2022.08.005 35963428

[B135] Moreira-Neto C. A. Bergeron S. Coblentz J. Zoroquiain P. Maloney S. Mastromonaco C. (2019). Optimizing optical coherence tomography and histopathology correlation in retinal imaging. Can. J. Ophthalmol., 54(2), 280–287. 10.1016/j.jcjo.2018.06.024 30975355

[B136] Morgan S. J. Elangbam C. S. Berens S. Janovitz E. Vitsky A. Zabka T. (2013). Use of animal models of human disease for nonclinical safety assessment of novel pharmaceuticals. Toxicol. Pathol. 41 (3), 508–518. 10.1177/0192623312457273 22968286

[B137] Moses T. Papadopoulou K. K. Osbourn A. (2014). Metabolic and functional diversity of saponins, biosynthetic intermediates and semi-synthetic derivatives. Crit. Rev. Biochem. Mol. Biol. 49 (6), 439–462. 10.3109/10409238.2014.953628 25286183 PMC4266039

[B138] Multani P. K. Modi R. Basu S. (2022). Pattern of recurrent inflammation following anti-tubercular therapy for ocular tuberculosis. Ocular Immunol. Inflamm. 30 (1), 185–190. 10.1080/09273948.2020.1772838 32644846

[B139] Nagarwal R. C. Kant S. Singh P. N. Maiti P. Pandit J. K. (2009). Polymeric nanoparticulate system: a potential approach for ocular drug delivery. J. Control. Release 136 (1), 2–13. 10.1016/j.jconrel.2008.12.018 19331856

[B140] Nair A. Greeny A. Nandan A. Sah R. K. Jose A. Dyawanapelly S. (2023). Advanced drug delivery and therapeutic strategies for tuberculosis treatment. J. Nanobiotechnology 21 (1), 414. 10.1186/s12951-023-02156-y 37946240 PMC10634178

[B141] Navarro V. J. Bonkovsky H. L. Hwang S.-I. Vega M. Barnhart H. Serrano J. (2013). Catechins in dietary supplements and hepatotoxicity. Dig. Dis. Sci. 58 (9), 2682–2690. 10.1007/s10620-013-2687-9 23625293 PMC3769469

[B142] Nawab A. Farooq N. (2015). Review on green tea constituents and its negative effects. J. Pharm. Innov. 4 (1), 21–24.

[B143] Ng T. K. Chu K. O. Wang C. C. Pang C. P. (2023). Green tea catechins as therapeutic antioxidants for glaucoma treatment. Antioxidants 12 (7), 1320. 10.3390/antiox12071320 37507860 PMC10376590

[B144] Nguta J. M. Appiah-Opong R. Nyarko A. K. Yeboah-Manu D. Addo P. G. A. (2015). Current perspectives in drug discovery against tuberculosis from natural products. Int. J. Mycobacteriology 4 (3), 165–183. 10.1016/j.ijmyco.2015.05.004 27649863

[B145] Nunes J. E. Duque M. A. de Freitas T. F. Galina L. Timmers L. F. Bizarro C. V. (2020). *Mycobacterium tuberculosis* shikimate pathway enzymes as targets for the rational design of anti-tuberculosis drugs. Molecules 25 (6), 1259. 10.3390/molecules25061259 32168746 PMC7144000

[B146] Oakenfull D. Sidhu G. S. (2023). “Saponins,” in Toxicants of plant origin, 97–142.

[B147] Oloyede H. O. B. Ajiboye H. O. Salawu M. O. Ajiboye T. O. (2017). Influence of oxidative stress on the antibacterial activity of betulin, betulinic acid and ursolic acid. Microb. Pathog., 111, 338–344. 10.1016/j.micpath.2017.08.012 28807773

[B148] Ozturk M. Chia J. E. Hazra R. Saqib M. Maine R. A. Guler R. (2021). Evaluation of berberine as an adjunct to TB treatment. Front. Immunol. 12, 656419. 10.3389/fimmu.2021.656419 34745081 PMC8563784

[B149] Pak J. Chen Z. J. Sun K. Przekwas A. Walenga R. Fan J. (2018). Computational modeling of drug transport across the *in vitro* cornea. Comput. Biol. Med., 92, 139–146. 10.1016/j.compbiomed.2017.11.009 29175100 PMC5766268

[B150] Pancu D. F. Scurtu A. Macasoi I. G. Marti D. Mioc M. Soica C. (2021). Antibiotics: conventional therapy and natural compounds with antibacterial activity—A pharmaco-toxicological screening. Antibiotics 10 (4), 401. 10.3390/antibiotics10040401 33917092 PMC8067816

[B151] Parvez M. K. Anjum N. (2022). Liver diseases and adverse reactions induced by medicinal herbs and herb-drug interactions. Gastroenterology and Hepatology Lett. 4 (1), 25–29. 10.18063/ghl.v4i1.280

[B152] Pasqualoto K. F. M. Ferreira E. I. (2001). An approach for the rational design of new antituberculosis agents. Curr. Drug Targets 2 (4), 427–437. 10.2174/1389450013348227 11732641

[B153] Patel P. Shastri D. Shelat P. Shukla A. (2010). Ophthalmic drug delivery system: challenges and approaches. Syst. Rev. Pharm. 1 (2), 113. 10.4103/0975-8453.75042

[B154] Patra A. K. (2012). An overview of antimicrobial properties of different classes of phytochemicals. Diet. phytochemicals microbes, 1–32. 10.1007/978-94-007-3926-0_1

[B155] Peña J. C. Ho W.-Z. (2017). Non-human primate models of tuberculosis. In Tuberculosis and the tubercle bacillus (pp. 163–176). 10.1128/9781555819569.ch8

[B156] Pérez-González G. L. Villarreal-Gómez L. J. Serrano-Medina A. Torres-Martínez E. J. Cornejo-Bravo J. M. (2019). Mucoadhesive electrospun nanofibers for drug delivery systems: applications of polymers and the parameters’ roles. Int. J. Nanomedicine 14 (null), 5271–5285. 10.2147/IJN.S193328 31409989 PMC6643962

[B157] Pikhtirova A. Pecka-Kiełb E. Zigo F. (2023). Antimicrobial activity of saponin-containing plants: review. J. Dairy Vet. Anim. Res. 2, 121–127. 10.15406/jdvar.2023.12.00336

[B158] Prajapati R. N. Bhushan B. Singh K. Chopra H. Kumar S. Agrawal M. (2024). Recent advances in pharmaceutical design: unleashing the potential of novel therapeutics. Curr. Pharm. Biotechnol. 25 (16), 2060–2077. 10.2174/0113892010275850240102105033 38288793

[B159] Prasad R. Singh A. Gupta N. (2021). Adverse drug reactions with first-line and second-line drugs in treatment of tuberculosis. Ann. Natl. Acad. Med. Sci. (India) 57 (01), 16–35. 10.1055/s-0040-1722535

[B160] Prusty G. Panda S. R. Behera R. R. Bhushan S. L. Rath A. Prusty P. K. (2024). “Natural product-based treatment for tuberculosis,” in Natural products for antibacterial drug development: recent advancement of computational approach (Springer), 79–95.

[B161] Quan D. Nagalingam G. Payne R. Triccas J. A. (2017). New tuberculosis drug leads from naturally occurring compounds. Int. J. Infect. Dis., 56, 212–220. 10.1016/j.ijid.2016.12.024 28062229

[B162] Rabaan A. A. Alhumaid S. Albayat H. Alsaeed M. Alofi F. S. Al-Howaidi M. H. (2022). Promising antimycobacterial activities of flavonoids against mycobacterium sp. drug targets: a comprehensive review. Molecules 27 (16), 5335. 10.3390/molecules27165335 36014572 PMC9415813

[B163] Ramsay E. del Amo E. M. Toropainen E. Tengvall-Unadike U. Ranta V.-P. Urtti A. (2018). Corneal and conjunctival drug permeability: systematic comparison and pharmacokinetic impact in the eye. Eur. J. Pharm. Sci., 119, 83–89. 10.1016/j.ejps.2018.03.034 29625211

[B164] Ramsay E. Hagström M. Vellonen K.-S. Boman S. Toropainen E. del Amo E. M. (2019). Role of retinal pigment epithelium permeability in drug transfer between posterior eye segment and systemic blood circulation. Eur. J. Pharm. Biopharm., 143, 18–23. 10.1016/j.ejpb.2019.08.008 31419586

[B165] Ribeiro A. Oliveira D. Cabral-Marques H. (2025). Curcumin in ophthalmology: mechanisms, challenges, and emerging opportunities. Molecules 30 (3), 457. 10.3390/molecules30030457 39942561 PMC11820683

[B166] Sabhapandit S. Gella V. Shireesha A. Thankachan L. Ismail M. Rao R. (2023). Ethambutol optic neuropathy in the extended anti-tubercular therapy regime: a systematic review. INDIAN J. Ophthalmol. 71 (3), 729–735. 10.4103/ijo.IJO_1920_22 36872667 PMC10229994

[B167] Sadeghi E. Imenshahidi M. Hosseinzadeh H. (2023). Molecular mechanisms and signaling pathways of Black cumin (Nigella sativa) and its active constituent, thymoquinone: a review. Mol. Biol. Rep. 50 (6), 5439–5454. 10.1007/s11033-023-08363-y 37155017

[B168] Sarangi A. Das B. S. Patnaik G. Sarkar S. Debnath M. Mohan M. (2021). Potent anti‐mycobacterial and immunomodulatory activity of some bioactive molecules of Indian ethnomedicinal plants that have the potential to enter in TB management. J. Appl. Microbiol. 131 (4), 1578–1599. 10.1111/jam.15088 33772980

[B169] Sasikumar K. Ghosh A. R. Dusthackeer A. (2018). Antimycobacterial potentials of quercetin and rutin against *Mycobacterium tuberculosis* H37Rv. 3 Biotech. 8 (10), 427. 10.1007/s13205-018-1450-5 30305996 PMC6162196

[B170] Saux N. L. MacDonald N. Dayneka N. (1997). Rifabutin ocular toxicity mimicking endophthalmitis. Pediatr. Infect. Dis. J. 16 (7), 716–718. 10.1097/00006454-199707000-00018 9239780

[B171] Semmar N. (2024). “Nitrogen-containing compounds,” in Secondary metabolites in plant stress adaptation: analytic space of secondary metabolites. Editor Semmar N. (Springer International Publishing), 111–152. 10.1007/978-3-031-52595-7_6

[B172] Sen S. (2017). “Pathogenesis and pathology of ocular tuberculosis,” in Ocular tuberculosis. Editors Kumar A. Chawla R. Sharma N. (Springer International Publishing), 7–15. 10.1007/978-3-319-57520-9_2

[B173] Shamsudin N. F. Ahmed Q. U. Mahmood S. Ali Shah S. A. Khatib A. Mukhtar S. (2022). Antibacterial effects of flavonoids and their structure-activity relationship study: a comparative interpretation. Molecules 27 (4), 1149. 10.3390/molecules27041149 35208939 PMC8879123

[B174] Sharma S. Kumar M. Sharma S. Nargotra A. Koul S. Khan I. A. (2010). Piperine as an inhibitor of Rv1258c, a putative multidrug efflux pump of *Mycobacterium tuberculosis* . J. Antimicrob. Chemother. 65 (8), 1694–1701. 10.1093/jac/dkq186 20525733

[B175] Sharma A. Thapa B. Lavaju P. (2011). Ocular tuberculosis: an update. Nepal. J. Ophthalmol. 3 (1), 52–67. 10.3126/nepjoph.v3i1.4280 21587325

[B176] Sharma A. Biharee A. Kumar A. Jaitak V. (2020). Antimicrobial terpenoids as a potential substitute in overcoming antimicrobial resistance. Curr. Drug Targets 21 (14), 1476–1494. 10.2174/1389450121666200520103427 32433003

[B177] Shastri D. H. Silva A. C. Almeida H. (2023). Ocular delivery of therapeutic proteins: a review. Pharmaceutics 15 (1), 205. 10.3390/pharmaceutics15010205 36678834 PMC9864358

[B178] Shirame S. P. Bhosale R. B. (2018). Green approach in click chemistry. Green Chem.

[B179] Shrestha A. Elliott S. Abasszade J. H. Wu K. Worland T. Simpson I. (2025). Drug-induced liver injury associated with turmeric and piperine: a case and review. Case Rep. Gastroenterology 19 (1), 96–106. 10.1159/000543679 39995754 PMC11850025

[B180] Si Z. Pethe K. Chan-Park M. B. (2023). Chemical basis of combination therapy to combat antibiotic resistance. JACS Au 3 (2), 276–292. 10.1021/jacsau.2c00532 36873689 PMC9975838

[B181] Sieniawska E. Sawicki R. Swatko-Ossor M. Napiorkowska A. Przekora A. Ginalska G. (2018). The effect of combining natural terpenes and antituberculous agents against reference and clinical *Mycobacterium tuberculosis* strains. Molecules 23 (1), 176. 10.3390/molecules23010176 29342972 PMC6017631

[B182] Singh P. K. Shukla P. (2012). Molecular modeling and docking of microbial inulinases towards perceptive enzyme–substrate interactions. Indian J. Microbiol. 52 (3), 373–380. 10.1007/s12088-012-0248-0 23997327 PMC3460115

[B183] Sizemore C. F. Schleif A. C. Bernstein J. B. Heilman C. A. (2012). The role of biomedical research in global tuberculosis control: gaps and challenges. Emerg. Microbes and Infect. 1 (1), 1–6. 10.1038/emi.2012.21 PMC3630913 26038420

[B184] Sulaiman I. I. Saad M. A. B. Bani-Saad A. A. Al-Khazaali Y. M. Al-Taie R. H. Al-Badri S. (2024). Challenges and insights in the diagnosis and management of orbital tuberculosis: a systematic review of 113 cases. Cureus 16 (9), e68976. 10.7759/cureus.68976 39385881 PMC11463888

[B185] Taldaev A. Terekhov R. Nikitin I. Zhevlakova A. Selivanova I. (2022). Insights into the pharmacological effects of flavonoids: the systematic review of computer modeling. Int. J. Mol. Sci. 23 (11), 6023. 10.3390/ijms23116023 35682702 PMC9181432

[B186] Taliou A. Zintzaras E. Lykouras L. Francis K. (2013). An open-label pilot study of a formulation containing the anti-inflammatory flavonoid luteolin and its effects on behavior in children with autism spectrum disorders. Clin. Ther. 35 (5), 592–602. 10.1016/j.clinthera.2013.04.006 23688534

[B187] Taskar P. Tatke A. Majumdar S. (2017). Advances in the use of prodrugs for drug delivery to the eye. Expert Opin. drug Deliv. 14 (1), 49–63. 10.1080/17425247.2016.1208649 27441817 PMC7337245

[B188] Testi I. Agrawal R. Mehta S. Basu S. Nguyen Q. Pavesio C. (2020). Ocular tuberculosis: where are we today? Indian J. Ophthalmol. 68 (9), 1808–1817. 10.4103/ijo.IJO_1451_20 32823397 PMC7690544

[B189] Thakur M. Mergel K. Weng A. von Mallinckrodt B. Gilabert-Oriol R. Dürkop H. (2013). Targeted tumor therapy by epidermal growth factor appended toxin and purified saponin: an evaluation of toxicity and therapeutic potential in syngeneic tumor bearing mice. Mol. Oncol., 7(3), 475–483. 10.1016/j.molonc.2012.12.004 23298730 PMC5528469

[B190] Thapliyal R. Maru G. (2001). Inhibition of cytochrome P450 isozymes by curcumins *in vitro* and *in vivo* . Food Chem. Toxicol. 39 (6), 541–547. 10.1016/s0278-6915(00)00165-4 11346483

[B191] Tizard I. R. (2020). Adjuvants and adjuvanticity. Vaccines veterinarians 75. 10.1016/B978-0-323-68299-2.00016-2

[B192] Toda R. Kawazu K. Oyabu M. Miyazaki T. Kiuchi Y. (2011). Comparison of drug permeabilities across the blood–retinal barrier, blood–aqueous humor barrier, and blood–brain barrier. J. Pharm. Sci., 100(9), 3904–3911. 10.1002/jps.22610 21638281

[B193] Wang R. Gao Y. Liu A. Zhai G. (2021). A review of nanocarrier-mediated drug delivery systems for posterior segment eye disease: challenges analysis and recent advances. J. drug Target. 29 (7), 687–702. 10.1080/1061186X.2021.1878366 33474998

[B194] Wang Y. Duan H. Zhang Z. Chen L. Li J. (2024). Research progress on the application of natural medicines in biomaterial coatings. Materials 17 (22), 5607. 10.3390/ma17225607 39597430 PMC11595593

[B195] Wu Y. Tao Q. Xie J. Lu L. Xie X. Zhang Y. (2023). Advances in nanogels for topical drug delivery in ocular diseases. Gels 9 (4), 292. 10.3390/gels9040292 37102904 PMC10137933

[B196] Xie Y. Yang W. Tang F. Chen X. Ren L. (2015). Antibacterial activities of flavonoids: structure-activity relationship and mechanism. Curr. Med. Chem. 22 (1), 132–149. 10.2174/0929867321666140916113443 25245513

[B197] Yoon M. S. Lee J. M. Jo M. J. Kang S. J. Yoo M. K. Park S. Y. (2025). Dual-drug delivery systems using hydrogel–nanoparticle composites: recent advances and key applications. Gels 11 (7), 520. 10.3390/gels11070520 40710682 PMC12294678

[B198] Zernii Y. Baksheeva E. Iomdina N. Permyakov E. Philippov P. Zamyatnin A. (2016). Rabbit models of ocular diseases: new relevance for classical approaches. CNS and Neurological Disord. - Drug Targets 15 (3), 267–291. 10.2174/1871527315666151110124957 26553163

[B199] Zhao L. Song J. Du Y. Ren C. Guo B. Bi H. (2023). Therapeutic applications of contact lens-based drug delivery systems in ophthalmic diseases. Drug Deliv. 30 (1), 2219419. 10.1080/10717544.2023.2219419 37264930 PMC10240982

[B200] Zhou H. Wang W. Cai L. Yang T. (2023). Potentiation and mechanism of berberine as an antibiotic adjuvant against multidrug-resistant bacteria. Infect. Drug Resist. 16 (null), 7313–7326. 10.2147/IDR.S431256 38023403 PMC10676105

[B201] Zimmerman A. Boyd R. F. Yekkala K. (2021). Correlations between retinal optical coherence tomography and histopathology in preclinical safety assessment of ocular therapies. Toxicol. Pathol. 49 (3), 528–536. 10.1177/0192623321989646 33568004

[B202] Zucca G. Vigani B. Valentino C. Ruggeri M. Marchesi N. Pascale A. (2025). Chondroitin sulphate-chitosan based nanogels loaded with Naringenin-β-Cyclodextrin complex as potential tool for the treatment of diabetic retinopathy: a formulation study. Int. J. Nanomedicine 20, 907–932. 10.2147/IJN.S488507 39867306 PMC11766310

